# Political ideology and moral dilemmas in public good provision

**DOI:** 10.1038/s41598-023-29512-0

**Published:** 2023-02-13

**Authors:** Laura C. Hoenig, Ruthie Pliskin, Carsten K. W. De Dreu

**Affiliations:** 1 Department of Social, Economic, and Organisational Psychology, Leiden University, Leiden, The Netherlands; 2 Center for Research in Experimental Economics and Political Decision Making, University of Amsterdam, Amsterdam, The Netherlands

**Keywords:** Human behaviour, Psychology and behaviour

## Abstract

Individuals often face dilemmas in which non-cooperation serves their self-interest and cooperation favors society at large. Cooperation is often considered the moral choice because it creates equality and fairness among citizens. Accordingly, individuals whose political ideology attaches greater value to equality than to agency and self-reliance should not only cooperate on more rather than less efficient public goods, but also more on public goods from which individuals benefit equally rather than unequally. We examine this possibility by comparing ideologically left-leaning and right-leaning individuals’ cooperation on multiple public goods that varied in efficiency and (in)equality in returns. We find that left-leaning individuals cooperate more than right-leaning ones, but only on public goods that benefit everyone equally, and not more on public goods that generate inequalities. Left-leaning individuals also trust and expect others to cooperate more on equal- versus unequal-returns public goods, while self-identified right-leaning individuals do not differentiate between these. Interestingly, ideology does not predict which public good is deemed more morally appropriate to cooperate on. Results combined specify when and why self-identified leftists can(not) be expected to cooperate more than rightists and reveal how moral decision-making depends on structural elements of the public good provision problems that citizens face.

## Introduction

Individuals in contemporary societies continuously face social dilemmas in which cooperation is in the best interest of the society, yet non-cooperation serves the individual best. For example, paying income tax is individually costly yet doing so allows societies to create and maintain public goods like collective healthcare and accessible education from which all citizens can benefit. Accordingly, cooperation is often construed as the morally right thing to do^1–4^, though this may be conditioned by the individual’s prevailing moral and political ideology—their ‘set of beliefs about the proper order of society, and how to achieve it’^5^. In fact, individuals who self-identify as political leftists attach moral value to equality^6–14^, which could make them more likely to cooperate on public goods that facilitate the equal distribution of resources, as in the examples provided above. Indeed, there are some indications that leftists tend to cooperate more in social dilemmas than individuals who self-identify as political rightists^15–21^. Self-identified rightists, in contrast, value personal agency and self-reliance and tend to be more accepting of wealth and status differences within and between societies^12,14,22^. Accordingly, researchers have demonstrated that rightists are more likely to freeride^23,24^ and less likely to make personally costly contributions to public goods, especially when interacting with strangers^25,26^.

But not all research has identified such differences. In fact, ideological differences have failed to emerge in several examinations of cooperation^26–29^, or appeared small and inconsistent^17^. One possible reason may be because the notion that leftist political ideology encourages cooperation more than rightist political ideology assumes – implicitly or explicitly – that cooperation creates equality, and that non-cooperation and free-riding creates or amplifies wealth *in*equalities. This is, however, not always the case. For example, paying income tax to support investments in neighborhoods often benefits those individuals residing in richer neighborhoods more than those living in poorer neighborhoods. In such situations, leftist individuals—who attach moral value to fairness and equality more than rightists—may be comparatively aversive of contributing to public goods that create and amplify inequalities within society. This is, in parts, because leftists more than rightists tend to be more motivated to express empathy, and to extend empathy to more distant others^6,14,30,31^.

**Figure 1:**
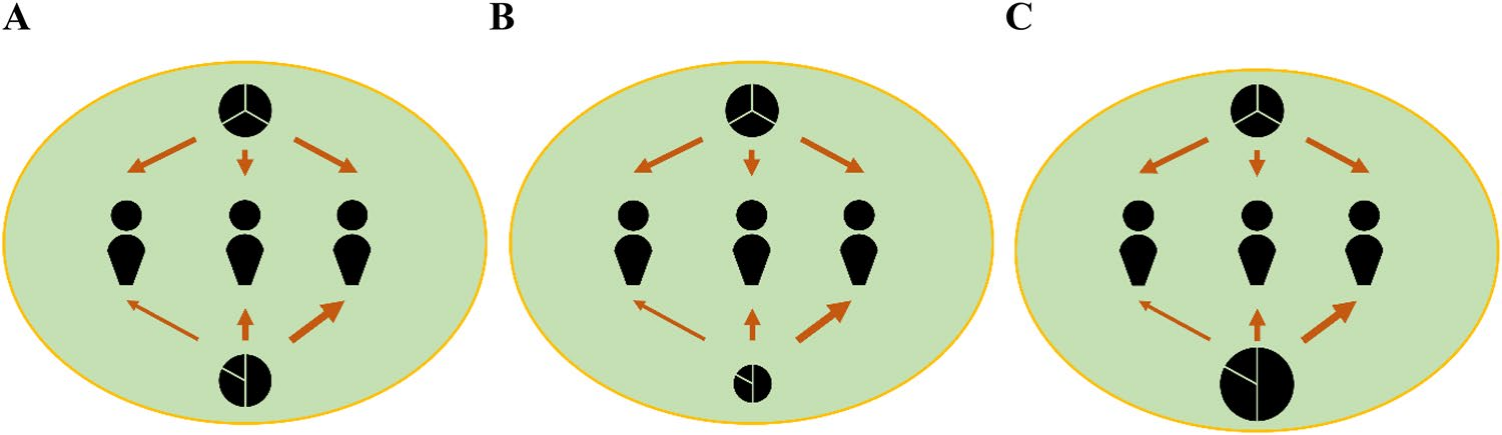
Unequal returns in a multiple-public goods game. Three individuals can contribute out of a personal endowment to two public goods depicted as black circles, that vary in their efficiency, depicted by the circles’ surfaces (**B, C**) and that provide (un)equal returns to group members, depicted as the part of the circle and the thickness of the arrows (**A**), where the equal-returns public good is more efficient than the unequal-returns public good (no decision conflict), (**B**) or where the equal-returns public good is less efficient than the unequal returns public good (decision conflict) (**C**).

Whereas (in)equality in returns from public goods may thus be an important factor moderating the impact of political ideology on public good provision, evidence for this possibility is lacking. To fill this void, we examined here how ideological self-identification affects cooperation in multiple-public goods provision problems. We focus on situations in which individuals can cooperate on several public goods, with some providing equal returns to all group members and others providing unequal returns (Fig. 1A). In line with the general literature on inequality aversion and as a ‘manipulation check’ for our paradigm, we expect people to cooperate more on public goods that provide equal rather than unequal returns (Hypothesis 1a). Importantly, cooperation is partially grounded in attaching moral value to equality, and we expected this to be more prominent among left-leaning individuals. Accordingly, and fitting research linking political ideology with moral norms^14,32^, we expected this effect of (in)equality in returns to be stronger among self-identified left-leaning individuals than among right-leaning individuals (Hypothesis 1b). We note that evidence for these hypotheses would resonate with the general observation that political ideology sometimes does impact cooperation^17,19,33^ and sometimes does not^26,27,29^, and fit previous work revealing that holding a rightist (vs. leftist) ideology attenuates negative responses to inequality^6,34,35^.

In addition to how public good returns are distributed, some public goods are more efficient than others—they are personally less costly to cooperate on and collectively more beneficial (Fig. 1B, C). In our studies, we manipulated public good efficiency orthogonally to the public good’s (in)equality in returns. We expected people to cooperate more on more rather than less efficient public goods (Hypothesis 2). Although we had no a priori reason to expect the impact of public good efficiency to be moderated by political ideology, we suspected that left-leaning more than right-leaning individuals may experience an internal moral dilemma when public goods that provide equality are less efficient than the public goods that create or amplify inequality (Fig. 1C). Put differently, when equality and efficiency of public goods are ‘misaligned’, political ideology may impact how much and on which public goods individuals cooperate.

Hypotheses on cooperation thus far were grounded in the notion that people with different political orientations differ in the moral value they attach to equality. There may be an additional—or even alternative—mechanism related to what people expect others to do. Specifically, because cooperation is potentially exploitable by others’ free-riding, cooperation also depends on the degree to which people expect (i.e., trust) others to cooperate or not^36,37^. Hence, leftists may cooperate more because they hold more positive, benign beliefs about the behavior of others^38,39^, whereas rightists view the world as a more dangerous^38,40–42^ or hierarchical place^43^. Indeed, leftists cooperated more in trust games than rightists^33^, and trust partially mediates the relationship between ideology and cooperation in two-person public good games^17^ (but see^26^). Possibly, left-leaning more than right-leaning individuals expect others to cooperate, and this makes cooperation a comparatively more attractive course of action. This is Hypothesis 3.

## Methods summary and results

### Ideology, cooperation, and trust

Hypotheses were tested by combining data from three experiments reported in Hoenig et al. (2023)^44^ with a total *N* = 735. Participants were recruited via Prolific Academic (http://www.prolific.co) and resided in either the UK or the US. Procedures and materials were nearly identical across experiments, and collapsing across datasets allowed us to obtain sufficient statistical power to test effects for political ideology on cooperation and expectations alone and in interaction with key properties of the multiple-public goods problem with which participants were confronted (*Methods*). Political ideology was measured on a 7-point Likert scale, asking “On a left–right political spectrum, how would you describe your political orientation?” and ranging from “extreme left” to “extreme right” (for more information on the distribution of political ideology in our sample, see Fig. S1 in the supplement). For the multiple-public goods game, individuals were organized in groups of three and given the possibility to contribute none, all, or part of a personal endowment to two public goods, one providing equal returns and the other providing unequal returns (henceforth ‘equal’ public good and ‘unequal’ public good, respectively) (Fig. 1A–C). Group members were assigned to be the low, intermediate, or high beneficiary from the unequal public good, but this between-subjects factor did not qualify the results for ideology and is thus further disregarded (see *Methods*).

For both public goods, free-riding maximized personal earnings, and contributing one’s full endowment maximized collective earnings. Participants made their contribution decisions in three blocks (order counterbalanced across participants). In one Block, the equal and unequal public goods were equally efficient; in one Block the equal public good was more efficient than the unequal public good, and in one Block, the equal public good was less efficient than the unequal public good (*Methods*). In this last Block, avoiding inequality required collective benefits to be sacrificed, and maximizing collective benefits implied group members who are equal in wealth ex ante will differ in wealth ex post. Accordingly, an individual’s earnings were the sum of (i) the remainder of their endowment after cooperating on the equal and unequal public goods, and (ii) their shares of the return from the equal and unequal public goods.

We ran all following analyses twice—once as reported here, and once controlling for participants’ country of residence. Adding this control did not alter our findings in any meaningful way and is henceforth ignored (see Tables S19-S28 in the supplement).

As reported in Hoenig et al. (2023), across efficiency levels, participants contributed 32% (*SD* = 2.98) of their endowment to the equal-returns public good, significantly more than the 22% (*SD* = 2.69) they contributed to the unequal-returns public good (β = − 0.96, 95% CI = [ − 1.05,  − 0.88], *p* < 0.001; see Table S2), thereby confirming our Hypothesis 1a. When taking into account levels of efficiency, however, participants only cooperated relatively more on the equal public good when it exceeded (40%, *SD* = 3.16 versus 12%, *SD* = 1.77; Table S3) or met the efficiency of the unequal public good (34%, *SD* = 3.95 versus 20%, *SD* = 2.39). When the equal public good was less efficient than the unequal public good, participants cooperated more on the unequal public good (22%, *SD* = 2.50 versus 35%, *SD* = 3.20).

**Figure 2:**
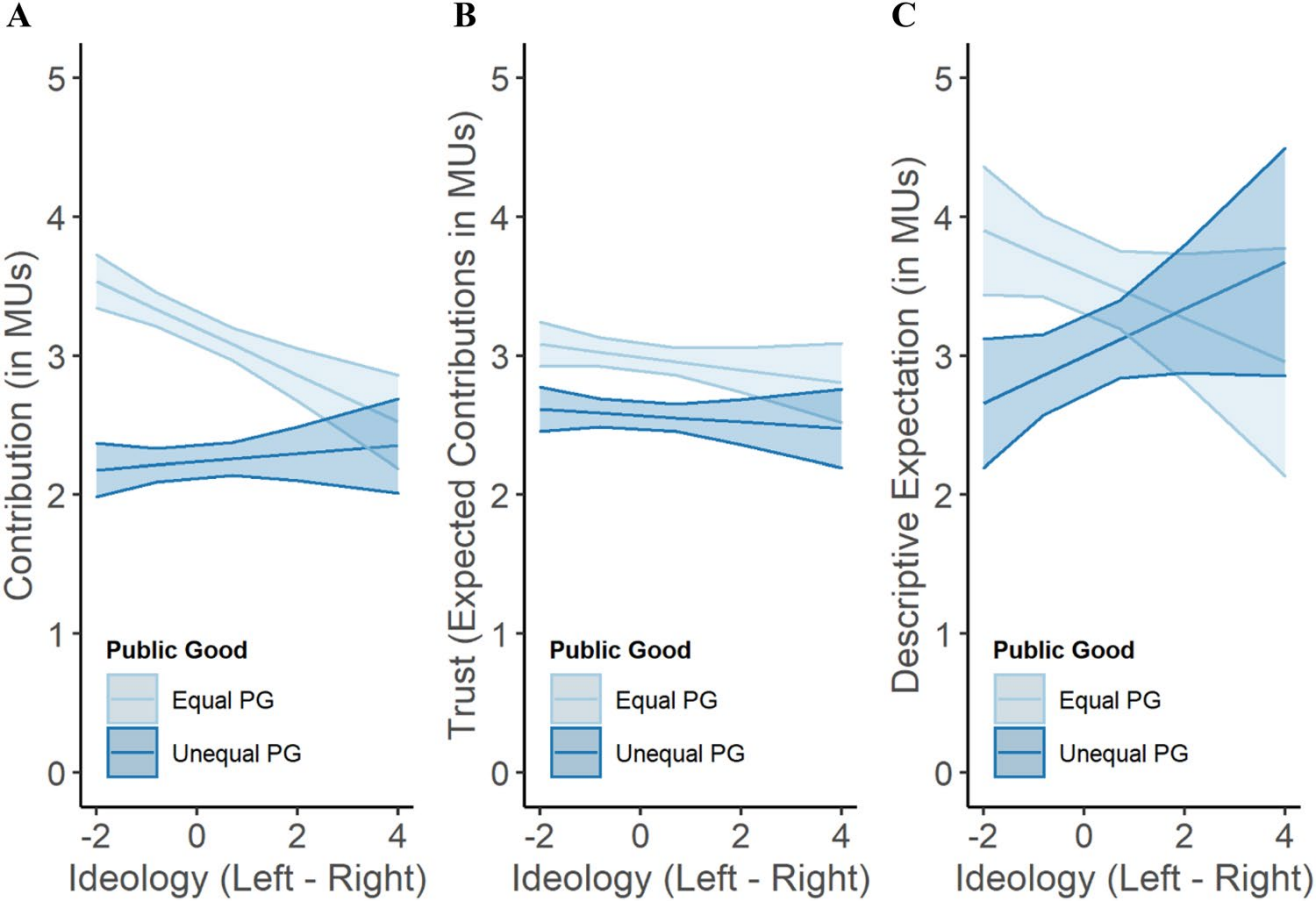
Ideology predicts cooperation, trust, and descriptive expectations. Cooperation on the equal and unequal public goods by leftists and rightists (**A**). Left- and right-leaning participants’ trust, measured as expectations of others’ contributions to the equal and unequal public good (**B**). Ideology predicts descriptive expectations about cooperation on the respective public goods (**C**). Specifically, left-leaning individuals expect others to contribute more to the equal than to the unequal public good, while right-leaning ones expect others not to differentiate between the two public goods.

Overall, right-leaning participants did not cooperate significantly less than left-leaning participants (Ideology: β =  − 0.07, 95% CI = [ − 0.14, 0.00], *p* = 0.061; Table S2). As predicted in Hypothesis 1a, they did, however, cooperate less than left-leaning participants on public goods that secured equality—but not less than them on public goods that created inequality (Ideology x Public Good: β = 0.20, 95% CI = [0.13, 0.26], *p* < 0.001; Table S4 Fig. 2A). Of note is that this general pattern holds when the equal public good is, relative to the unequal public good, equally or more efficient (Ideology x Public Good x Condition (equal PG = unequal PG): β = 0.30, 95% CI = [0.14, 0.45], *p* < 0.001; Ideology x Public Good x Condition (equal PG > unequal PG): β = 0.43, 95% CI = [0.28, 0.59], *p* < 0.001; see Table S4 and Fig. S3 in the supplement). When the equal public good was less efficient, both left- and right-leaning participants contributed more to the more efficient unequal-returns public good (see Fig. S2A-C in the supplement for more details on Johnson-Neyman intervals that indicate where the simple slopes are significant in the interaction of political ideology and public good in this and the following analyses).

Results for trust in others’ cooperation largely mirrored the patterns observed for own cooperation (Fig. 2B; Tables S5-8). Participants trusted others to cooperate more on the equal rather than unequal public goods (30%, *SD* = 2.70 versus 26%, *SD* = 2.66; β =  − 0.42, 95% CI = [ − 0.44,  − 0.41], *p* < 0.001; Table S6). Political ideology did not relate to overall trust (β =  − 0.04, 95% CI = [ − 0.10, 0.03], *p* = 0.314), but, in line with the pattern observed for own cooperation, left-leaning participants trusted others to cooperate more on the equal public good than did right-leaning ones (β = 0.02, 95% CI = [0.01, 0.03], *p* < 0.001); for the unequal public good no such difference was observed (Table S8). Again, this general pattern holds when the equal public good is, relative to the unequal public good, more efficient, and not when the equal public good meets or falls short of the unequal public good efficiency (Table S9; see Fig. S3 in the supplement). We did not find trust to mediate the relationship between political ideology and cooperation (see supplementary information; Tables S10-13).

### Ideology and norms of cooperation

One key mechanism underlying leftists' tendency to cooperate more than rightists is that public goods provide equal returns and thus create an even distribution of wealth among group members. The moral value attached to equality was reflected in both own cooperation and trust regarding others’ cooperation. Possibly, leftists, more than rightists, follow a norm that cooperating towards equality is the morally appropriate thing to do. To examine this possibility, we performed a follow-up study. We tested differences in normative and descriptive expectations^45^ based on ideology, public good, and their interaction (participant gender and the position of the target were included as covariates; see Tables S17 and S18).

In the study, both norms were measured by asking participants only about the condition in which both public goods had the same efficiency (i.e., when equality and inequality needed to be traded off). To measure normative expectations, we adapted the Krupka-Weber method^46^ and asked participants which contributions they found “socially appropriate”; to measure descriptive expectations, participants were asked to estimate how they think other participants would, in actuality, contribute to the public goods.

In line with the patterns observed in our previous study, we found that (uninvolved) participants found it more socially appropriate for, and expected, participants in the multiple-public goods game to cooperate more on the equal public good (normative expectations: 47%, *SD* = 2.39; descriptive expectations: 36%, *SD* = 2.24) than on the unequal public good (normative expectations: 27%, *SD* = 2.11; β =  − 2.03, 95% CI = [ − 2.35  − 1.70], *p* < 0.001; descriptive expectations: 30%, *SD* = 2.33; β =  − 0.59, 95% CI = [ − 0.91,  − 0.27], *p* < 0.001).

Whereas political ideology related to neither normative expectations (β =  − 0.10, 95% CI = [ − 0.23, 0.03], *p* = 0.148) nor descriptive expectations of overall cooperation (β = 0.01, 95% CI = [ − 0.14, 0.16], *p* = 0.936), left-leaning participants expected others to cooperate more on the equal public good than did right-leaning participants; left- and right-leaning participants did not differ in their descriptive expectations regarding cooperation on the unequal public good (β = 0.33, 95% CI = [0.08, 0.58], *p* = 0.011; see Fig. 2C).

Whereas ideology was related to different descriptive expectations, it did not significantly relate to normative expectations of cooperation on the different public goods (β = 0.19, 95% CI = [ − 0.07, 0.44], *p* = 0.155). Accordingly, it appears that both left- and right-leaning participants deemed it socially appropriate to cooperate more on the equal public good than on the unequal public good (Table S17).

## Discussion

Our experiments confirm some of the few existing findings on a relationship between ideology and cooperation, while clarifying why such findings have not always been consistent and contributing to the literature with four important points. Firstly, the general relationship between political ideology and cooperation did not reach statistical significance. Overall cooperation trends into the direction shown by Grünhage and Reuter in a standard public goods game^15^, but we cannot confidently verify their finding of higher overall cooperation by leftists unless we also examine on *which* public goods leftists and rightists cooperate. Whereas cooperation on a public good that provides unequal returns did not differ per ideology, left-leaning individuals cooperated far more than right-leaning ones on an equal-returns public good (Hypothesis 1b). Moreover, we obtained some evidence that this effect is limited to situations in which the equal public good meets or exceeds the efficiency of an alternative, unequal public good. When the unequal-returns public good provides greater collective welfare, something core to leftists’ values^7,23,47^, leftists’ preference for efficiency overrides their general preference for equality (also see ^48,49^).

Earlier work showed that cooperation in economic games may be explained by the trust that people have that their fellow group members will also cooperate (in line with^17,50,51^; Hypothesis 3). Left-leaning individuals in our sample did trust more than right-leaning ones that others would cooperate on a public good that allows both equal returns and high efficiency. The effect does not seem to be very strong, however, and we did not find mediation of cooperation through trust (also see^16,17^). Norms may provide additional insight into the relationship of ideology and cooperation, as general population expectations contain more variation than individual beliefs.

Perhaps not surprisingly, political ideology—a conglomerate of beliefs about the proper order of society and how to achieve it—has been shown to relate to norm abiding behavior and moral norms^14,32^. To the authors’ knowledge, not much work has investigated the relationship of ideology and social norms in social dilemmas. We find that ideology neither predicts normative nor descriptive expectations of *overall* cooperation when both public goods are of same efficiency. Notably, ideology neither predicts normative expectations of cooperation on the respective public goods—both left- and right-leaning individuals deem it morally appropriate to cooperate more on the public good providing equal returns rather than unequal returns. Left- and right-leaning individuals do, however, carry different descriptive expectations about how others are going to behave in actuality. Left-leaning individuals expect that others will cooperate more on equal public goods than on unequal public goods, whereas right-leaning ones do not hold different expectations for the two public goods. If anything, the latter expect others to contribute relatively more to the unequal public good than to the equal one.

Connecting our findings across studies suggests that leftists’ behavior seems to be consistent with their trust towards fellow group members, as well as what they find morally appropriate and expect other people to do in general. In other words, when inequality and equality must be traded off at constant efficiency, leftists cooperate more on the equal public good than on the unequal public good, and this is in line with their trust, descriptive expectations, and normative expectations.

The combined findings suggest further that rightists may not be consistent in their behavior and norms when inequality and equality must be traded off at constant efficiency. Right-leaning individuals in our sample do not differentiate cooperation on the equal public good from the unequal public good, and they do not expect other people to do so. Both right-leaning individuals inside and outside of the multiple-public goods game expect (i.e., trust) others to cooperate relatively more on unequal public goods, speaking to previous findings that rightists see the world as a more dangerous place^38,40–42^ or, as presented in recent findings, a more hierarchical place than leftists do^43^. However, uninvolved right-leaning individuals do deem it morally appropriate to cooperate more on the equal public good than on the unequal public good, as do left-leaning ones. This pattern indicates the possibility that rightists’ cooperation behavior may be driven by (descriptive) expectations rather than by normative expectations, while leftists’ cooperation may be driven by both descriptive and normative expectations. Right-leaning individuals being inconsistent in their normative expectations and their behavior appears puzzling, given the fact that past research has nearly unanimously found rightists to be more norm abiding than leftists^14,52,53^. Relatedly, (right wing) authoritarianism has been linked with increased norm adherence^54^ and, on the flip side, aversion towards norm violations^33^.

These findings indicate to us that it is a relevant avenue to identify the (types of) social norms at play amongst leftists and rightists, to unravel when and why they (do not) cooperate in social dilemmas. Future research may further investigate this mechanism and whether it holds in additional national contexts. More specifically, it may be interesting for future work to investigate whether our findings hold in populations that are less polarized or where different cultural norms are at play.

### Conclusion

Cooperation is often considered the morally right behavior in public good problems. We propose that the literature has neglected two important qualifications of this claim: Behavior, trust, and norms of cooperation vary based on context and the individuals’ political ideology, which shapes some of their moral convictions^55,56^. Scholars have presented divergent findings as to whether ideology matters for cooperation, with strong proponents on both sides of the debate. The present research may provide one possible explanation why related findings to date have been inconclusive: The effect of ideology on the willingness to cooperate depends on the context and, more specifically, on features of the public good that individuals cooperate on and that affect collective outcomes. With equality of returns and efficiency, we present two key features that can vary in public goods. At the same time, equality and efficiency concerns are central to many issues at the heart of divides between ideological leftists and rightists. The novel framework of the multiple-public goods game allows us to show how left-leaning and right-leaning individuals trade off equality and efficiency concerns in a social dilemma, and how this affects their levels of cooperation. As relevant avenues to further understand the mechanisms underlying this behavior, we suggest trust as well as normative expectations of others, which seem to reflect individuals’ behavior.

## Methods

### Public good provision experiment

#### Participants and experimental procedure

The public good provision experiment involved data from three experiments (Exp. 2–4 in Hoenig et al., 2022). Experimental protocols and hypotheses were pre-registered (Exp. 1: #39523 at https://aspredicted.org/IIV_LVO; Exp. 2: #47435 at https://aspredicted.org/OAL_YEYl; Exp. 3: #62406 at https://aspredicted.org/V3M_32L) and received ethics approval from the Psychology Research Ethics Committee at Leiden University (CEP; 2020–03-30-R.Pliskin-V1-2344; 2020–09-01-R.Pliskin-V1-2595; 2021–03-02-R.Pliskin-V2-2928). All methods were carried out in accordance with CEP guidelines as well as the Netherlands Code of Conduct for Research Integrity. Participants gave informed consent before participating and received full debriefing upon completion of the study. The studies were incentivized and involved no deception. Including the average baseline payment of 3.87 GBP/ 4.94 USD and earnings from the various decision-tasks, participants earned on average a total of 9.46 GBP/11.78 USD. Experiments were programmed in Qualtrics (Qualtrics, Provo, UT) and implemented online via Prolific Academic (http://www.prolific.co) with participants based in the UK or USA (total *N* = 735 participants; *M*_age_ = 28.67 years, *SD* = 6.95; 425 women, 5 non-binary or third gender, 7 not indicated). The design was the same across the three experiments, with only minor variations that did not yield statistically-significant differences (for more information, see the supplement). Accordingly, we present averaged data across all experiments.

#### Measures and Statistical Analyses

Across experiments, political ideology was measured on a 7-point Likert scale, asking “On a left–right political spectrum, how would you describe your political orientation?” and ranging from “extreme left” to “extreme right.” In addition, we measured social and economic ideology for exploratory purposes. Upon assuring that, for US residents, the left–right measure correlated highly with the liberal-conservative measure (Pearson *r*(4120) = 0.83, *p* < 0.001), we chose to use the identical left–right measure across countries and studies to ensure comparability, and disregarded the other measures further on. Political ideology was assessed either one week before the actual public good provision task (Experiments 1 and 2) or right after engaging in the decision-making (Experiment 3). In all cases, the measure was embedded in a series of survey items about a variety of topics that were otherwise irrelevant to the current project (see pre-registrations for more details).

For the public good provision task, participants read that they would be paired with two other participants to form a group of three individuals. They read that each group member would make decisions that would influence their personal earnings as well as those of the other two group members. Following task instructions and comprehension questions, participants made a series of contribution decisions in various multiple-public goods scenarios (see supplement Figure S1 in Hoenig et al., 2023, for visuals). Specifically, for each decision trial, participants received an endowment of 10 Monetary Units (MUs) and were instructed how they could contribute to two public goods. We explained that contributions would be deducted from their endowment yet would provide a ‘return on investment’ to each of the individuals in their group, themselves included. Each trial always contrasted an equal- and an unequal-returns public good. The calculation of payoffs was explained to participants in a stylized form to ensure understandability and reduce demand characteristics. Participants were instructed that each group member would receive one third of the payoffs from the equal-returns public good. Regarding the unequal public good, the example provided depicted the low beneficiary earning one sixth, the intermediate beneficiary earning one third, and the high beneficiary earning half of the returns. During the actual decision trials, differences between the beneficiaries were smaller, as explained in the following. For each of the two public goods, efficiency was operationalized at the range of 0 < MPCR < 1 (Marginal Per Capita Return; here with *n* = 3 group members). Accordingly, it was always individually rational not to invest anything in either public good (viz. *free-riding*), and it was always collectively rational to invest one’s entire endowment in one or both public goods.

A public good with a multiplier of 1.5 would thus provide each individual with 0.50 per MU contributed by oneself and the other individuals. For the unequal public good, we varied returns in such a way that one individual would receive the lowest return, one would receive the highest return, and the third would receive an intermediate return. For example, from an unequal public good with an overall efficiency of 1.5, one group member (the low beneficiary) received 0.43MU from each MU contributed (by themselves and the other two group members). An intermediate beneficiary in this case received 0.50MU per MU contributed, and a high beneficiary received 0.56 per MU contributed. Participants always saw on the decision screen how much one contributed MU would mean to them and their group members in their returns (e.g., for a low beneficiary: 0.43 to oneself, 0.50 to the intermediary, and 0.56 to the high beneficiary).

In total, participants made 9 decisions, organized in three blocks of three decisions each. Blocks varied the *relative* efficiency of the equal compared to the unequal-returns public good on which participants could cooperate. In one block, the unequal public good was as efficient as the equal public good, in another block, the unequal public good was relatively more efficient, and in one final block it was relatively less efficient. Within each block, we varied the *overall* efficiency of both public goods combined. Accordingly, across trials within each block, cooperating on the public goods provided small, medium, or high returns (see Table S1 in the supplement for more detail). Because this variable had no influence on our measures alone or in combination with other factors, it is further ignored. In addition, the specific position participants occupied in the unequal returns public good (being the low, intermediate, or high beneficiary) did not interact with our main independent variable, political ideology. Position was therefore included as a covariate in the analyses reported in the manuscript, and further ignored. Thus, the design for our experiment involved a 2 (equal versus unequal public good) × 3 (efficiency of the unequal relative to the equal public good: more, equal, or less) within-subjects factorial, with political ideology as a (centered) continuous predictor between-subjects.

Dependent variables were contributions to the (un)equal public good (range 0–10), and trust regarding others’ contributions. Trust was retrieved by asking individuals after they had indicated their own contribution on a particular trial as to how much they expected the other two individuals in their group contributed to each of the two public goods (in line with some earlier measures of trust in economic games^17,26^). Data were analyzed with mixed models, implemented with the R-package *lme4*. Political ideology was mean-centered and we performed, for each dependent variable, several mixed models with and without interaction effects (see model specifications in Tables S2-5 for cooperation, and Tables S6-13 for trust). Other levels of Relative Efficiency Condition are always compared against the condition when the unequal public good exceeds the equal public good efficiency, and the other Public Good level (unequal public good) is always compared against the equal public good as set level. Beneficiary position (low, intermediate, or high, with the latter two compared against the low position) and gender (with men and ‘other’ compared against women) were included as covariates. We also tested whether the effect of political ideology holds when controlling for social value orientation (SVO), and report the detailed results in the supplement (Tables S14-16). Results did not change and political ideology still had a unique effect on which public good participants cooperated on, as reported in the results above.

### Norm study

#### Participants and study procedure

We asked uninvolved participants to indicate both normative and descriptive expectations. By using uninvolved individuals, we hoped to reduce incentives to misreport and thereby aim at first-order expectations. We collected data from *N* = 120 participants from the USA and the UK via Prolific (*M*_age_ = 33.16, *SD* = 11.05; 40.8% women, 1 non-binary). The study received ethics approval (2021–03-02-R. Pliskin-V2-2928) and was pre-registered along with Experiment 3 (#62406 at https://aspredicted.org/V3M_32L). Participants read the information letter, indicated their informed consent, and, upon completing the study, received a written debriefing.

After completing an unrelated task, participants were presented with a paraphrased part 1 of the Krupka-Weber method for eliciting normative beliefs^46^ (see Fig. S4). This was followed by a detailed description of the multiple-public goods provision problem, in particular the situation in which the equal and unequal public good are equally efficient. Participants responded to comprehension checks about the rules of the game and proceeded to indicate, first, their normative expectations and, second, their descriptive expectations regarding all three beneficiaries when the efficiency of the equal and unequal public good were the same. Finally, participants responded to demographic questions and indicated their ideology.

#### Measures and statistical analyses

Normative expectations were measured by asking the participants which contributions they deemed “socially appropriate” by each of the three beneficiaries. Descriptive expectations were measured by asking them to indicate how they, on average, expected the three group members in the multiple-public goods game to allocate their endowments in actuality. We incentivized indications of descriptive expectations but not normative expectations. Ideology was measured as explained for Experiments 1–3.

To test for ideological differences in normative and descriptive expectations, we performed two mixed models each (Hypothesis 3; see model specifications in Tables S17 and S18). We included gender and the beneficiary position of the target as covariates.

## Supplementary Information

Supplementary Information.

## Data Availability

In line with the data regulations our institution is bound to, we will make the data available upon publication of the manuscript. The experiments were pre-registered on AsPredicted.org and data will be made accessible on OSF.io.

## References

[1] Curry OS, Mullins DA, Whitehouse H (2019). Is it good to cooperate? testing the theory of morality-as-cooperation in 60 societies. Curr. Anthropol..

[2] Tomasello M, Vaish A (2013). Origins of human cooperation and morality. Annu. Rev. Psychol..

[3] Capraro V, Rand DG (2018). Do the right thing: Experimental evidence that preferences for moral behavior, rather than equity or efficiency per se, drive human prosociality. Judgm. Decis. Mak..

[4] Biziou-Van-Pol L, Haenen J, Novaro A, Liberman AO, Capraro V (2015). Does telling white lies signal pro-social preferences?. Judgm. Decis. Mak..

[5] Erikson RS, Tedin KL (2019). American Public Opinion: Its Origins, Content, And Impact.

[6] Goudarzi S, Pliskin R, Jost JT, Knowles ED (2020). Economic system justification predicts muted emotional responses to inequality. Nat. Commun..

[7] Feldman, S. Values, ideology, and the structure of political attitudes. in *Oxford Handbook of Political Psychology* (eds. Sears, D. O., Huddy, L. & Jervis, R.) 477–508 (Oxford University Press, 2003).

[8] Fuchs, D. & Klingemann, H.-D. The Left/Right Schema. in *Continuities In Political Action: A Longitudional Study Of Political Orientations In Three Western Democracies* (eds. Jennings, M. K. & van Deth, J.) (Berlin: de Gruyter, 1989).

[9] Jost JT (2006). The end of the end of ideology. Am. Psychol..

[10] Jost JT, Federico CM, Napier JL (2008). Political ideology: its structure, functions, and elective affinities. Annu. Rev. Psychol..

[11] Knight, K. Liberalism and conservatism. in *Measures Of Political Attitudes* (eds. Robinson, J. P., Shaver, P. R. & Wrightsman, L. S.) 59–158 (San Diego, CAAcademic Press, 1999).

[12] Thorisdottir H, Jost JT, Liviatan I, Shrout PE (2007). Psychological needs and values underlying left-right political orientation: Cross-national evidence from eastern and western Europe. Public Opin. Q..

[13] Mikołajczak G, Becker JC (2019). What is (un)fair? political ideology and collective action. J. Soc. Polit. Psychol..

[14] Graham J, Haidt J, Nosek BA (2009). Liberals and conservatives rely on different sets of moral foundations. J. Pers. Soc. Psychol..

[15] Grünhage T, Reuter M (2022). Political orientation is associated with behavior in public-goods- and trust-games. Polit. Behav..

[16] Brewer, M. B., Buchan, N. R., Ozturk, O. D. & Grimalda, G. Parochial altruism and political ideology. *Polit. Psychol.* **0**, 1–14 (2022).

[17] Romano, A., Sutter, M., Liu, J. H. & Balliet, D. Political ideology, cooperation and national parochialism across 42 nations. *Philos. Trans. R. Soc. B Biol. Sci.* **376**, 20200146 (2021). 10.1098/rstb.2020.0146 PMC7934968 33611989

[18] Chaudhuri A, Sopher B, Strand P (2002). Cooperation in social dilemmas, trust and reciprocity. J. Econ. Psychol..

[19] Mansell J, Petersen MB (2022). Political ideologies as social strategies: does ideological variation predict behavioral variation in cooperative dilemmas?. Curr. Psychol..

[20] Raub W, Snijders C (1997). Gains, losses, and cooperation in social dilemmas and collective action: The effects of risk preferences. J. Math. Sociol..

[21] Claessens S, Fischer K, Chaudhuri A, Sibley CG, Atkinson QD (2020). The dual evolutionary foundations of political ideology. Nat. Hum. Behav..

[22] Iyer R, Koleva S, Graham J, Ditto P, Haidt J (2012). Understanding libertarian morality: The psychological dispositions of self-identified libertarians. PLoS ONE.

[23] Feldman S (1988). Structure and consistency in public opinion: the role of core beliefs and values. Am. J. Pol. Sci..

[24] Sheldon KM, Nichols CP (2009). Comparing democrats and republicans on intrinsic and extrinsic values. J. Appl. Soc. Psychol..

[25] Van Lange PAM, Bekkers R, Chirumbolo A, Leone L (2012). Are conservatives less likely to be prosocial than liberals? From games to ideology, political preferences and voting. Eur. J. Pers..

[26] Balliet D, Tybur JM, Wu J, Antonellis C, Van Lange PAM (2018). Political ideology, trust, and cooperation: In-group favoritism among republicans and democrats during a US national election. J. Conflict Resolut..

[27] Brandt MJ, Crawford JT (2020). Worldview conflict and prejudice. Adv. Exp. Soc. Psychol..

[28] Brandt MJ, Reyna C, Chambers JR, Crawford JT, Wetherell G (2014). The ideological-conflict hypothesis: Intolerance among both liberals and conservatives. Curr. Dir. Psychol. Sci..

[29] Wetherell GA, Brandt MJ, Reyna C (2013). Discrimination across the ideological divide: The role of value violations and abstract values in discrimination by liberals and conservatives. Soc. Psychol. Personal. Sci..

[30] Hasson Y, Tamir M, Brahms KS, Cohrs JC, Halperin E (2018). Are liberals and conservatives equally motivated to feel empathy toward others?. Personal. Soc. Psychol. Bull..

[31] Waytz A, Iyer R, Young L, Haidt J, Graham J (2019). Ideological differences in the expanse of the moral circle. Nat. Commun..

[32] Jost JT, Glaser J, Kruglanski AW, Sulloway FJ (2003). Political conservatism as motivated social cognition. Psychol. Bull..

[33] Claessens, S., Sibley, C. G., Chaudhuri, A. & Atkinson, Q. D. Cooperative and conformist behavioural preferences predict the dual dimensions of political ideology. *PsyArXiv* (2020). 10.1038/s41598-023-31721-6 PMC10039865 36966181

[34] Jost JT, Blount S, Pfeffer J, Hunyady G (2003). Fair market ideology: its cognitive and motivational underpinnings. Res. Organ. Behav..

[35] Napier JL, Jost JT (2008). Why are conservatives happier than liberals?. Psychol. Sci..

[36] Bogaert S, Boone C, Declerck C (2008). Social value orientation and cooperation in social dilemmas: A review and conceptual model. Br. J. Soc. Psychol..

[37] Van Dijk E, De Dreu CKW (2021). Experimental games and social decision making. Annu. Rev. Psychol..

[38] Duckitt J (2001). A dual-process cognitive-motivational theory of ideology and prejudice. Adv. Exp. Soc. Psychol..

[39] Duckitt J, Parra C (2010). Dimensions of group identification and out-group attitudes in four ethnic groups in New Zealand. Basic Appl. Soc. Psych..

[40] Altemeyer, B. *Right-Wing Authoritarianism*. (Univ. of Manitoba Press, 1983).

[41] van Leeuwen F, Park JH (2009). Perceptions of social dangers, moral foundations, and political orientation. Pers. Individ. Dif..

[42] Cook CL, Li YJ, Newell SM, Cottrell CA, Neel R (2018). The world is a scary place: Individual differences in belief in a dangerous world predict specific intergroup prejudices. Gr. Process. Intergr. Relations.

[43] Clifton JDW, Kerry N (2018). Belief in a dangerous world does not explain substantial variance in political attitudes, but other world beliefs do. Soc. Psychol. Personal. Sci..

[44] Hoenig, L. C., Pliskin, R. & De Dreu, C. K. W. *Equality and efficiency shape cooperation in multiple-public goods provision problems*. 10.31234/osf.io/re52c (2023) 10.1037/xge0001574 38647478

[45] Bicchieri, C. *Norms in the wild: How to diagnose, measure, and change social norms*. (Oxford University Press, 2016).

[46] Krupka EL, Weber RA (2013). Identifying social norms using coordination games: Why does dictator game sharing vary?. J. Eur. Econ. Assoc..

[47] Janoff-Bulman R (2009). To provide or protect: Motivational bases of political liberalism and conservatism. Psychol. Inq..

[48] Charness G, Rabin M (2002). Understanding social preferences with simple tests. Q. J. Econ..

[49] Engelmann D, Strobel M (2004). Inequality aversion, efficiency, and maximin preferences in simple distribution experiments. Am. Econ. Rev..

[50] Gerpott FH, Balliet D, Columbus S, Molho C, de Vries RE (2018). How do people think about interdependence? A multidimensional model of subjective outcome interdependence. J. Pers. Soc. Psychol..

[51] Van Lange PAM, Joireman J, Parks CD, Dijk EV, Van Dijk E (2013). The psychology of social dilemmas: A review. Organ. Behav. Hum. Decis. Process..

[52] Jost JT, Pelham BW, Sheldon O, Sullivan BN (2003). Social inequality and the reduction of ideological dissonance on behalf of the system: Evidence of enhanced system justification among the disadvantaged. Eur. J. Soc. Psychol..

[53] Claessens S, Chaudhuri A, Sibley CG, Atkinson QD, Osborne D, Sibley CG (2022). The Evolutionary Basis of Political Ideology. The Cambridge Handbook Of Political Psychology.

[54] Kessler, T. & Cohrs, J. C. The evolution of authoritarian processes: fostering cooperation in large-scale groups. *Gr. Dyn. Theory, Res. Pract.* **12**, 73–84 (2008).

[55] Reifen Tagar, M., Morgan, G. S., Halperin, E. & Skitka, L. J. When ideology matters: moral conviction and the association between ideology and policy preferences in the Israeli–Palestinian conflict. *Eur. J. Soc. Psychol.* **44**, 117–125 (2014).

[56] Skitka, L. J., Morgan, G. S. & Wisneski, D. C. Political orientation and moral conviction: A conservative advantage or an equal opportunity motivator of political engagement? in *Social Psychology And Politics* (eds. Forgas, J. P., Fiedler, K. & Crano, W. D.) 57–74 (Psychology Press, 2015).

