# Supplementary material for: Political ideology and moral dilemmas in public good provision
Source: Sci Rep. 2023 Feb 13;13:2519. doi: 10.1038/s41598-023-29512-0 (PMC9925786; doi:10.1038/s41598-023-29512-0)
Supplement: Supplementary file 1 — Supplementary Information. [file 41598_2023_29512_MOESM1_ESM.docx]

Supplementary Information:

Political Ideology and Moral Dilemmas
in Public Good Provision

Laura C. Hoenig^*a^

Ruthie Pliskin^a^

Carsten K. W. De Dreu^a,b^

^a^Department of Social, Economic, and Organisational Psychology
Leiden University

^b^Center for Research in Experimental Economics and Political Decision Making
University of Amsterdam

^*^Corresponding author
Email: l.c.hoenig@fsw.leidenuniv.nl

Contents

[1 Experiments 1-3 3](#_Toc126853018)

[1.1 Methods 3](#_Toc126853019)

[1.2 Measures 4](#_Toc126853020)

[1.2.1 Ideology 4](#_Toc126853021)

[1.2.2 Comprehension Questions 5](#_Toc126853022)

[1.3 Statistical Analyses 6](#_Toc126853023)

[1.4 Results 6](#_Toc126853024)

[1.4.1 Cooperation 6](#_Toc126853025)

[1.4.2 Trust 11](#_Toc126853026)

[1.4.2.1 Mediation of Ideology and Cooperation through Trust 14](#_Toc126853027)

[1.4.3 The role of Social Value Orientation 16](#_Toc126853028)

[2 Norm Study 19](#_Toc126853029)

[2.1 Measures 19](#_Toc126853030)

[2.1.1 Comprehension Questions 19](#_Toc126853031)

[2.2 Results 20](#_Toc126853032)

[2.3 Visuals of Norm Study 22](#_Toc126853033)

[3 Main Analyses controlling for Country of Residence 26](#_Toc126853034)

[3.1 Cooperation 26](#_Toc126853035)

[3.2 Trust 29](#_Toc126853036)

[3.3 Norms 32](#_Toc126853037)

[4 Supplement References 34](#_Toc126853038)

# 1 Experiments 1-3

## 1.1 Methods

Data were collected at two time points for Experiments 1 and 2, and at one time point for Experiment 3, which did not influence the results. At the second time point (for Experiments 1 and 2), participants completed three blocks (i.e., Conditions) of the multiple-public goods game: The unequal public good either fell short of, met, or exceeded the efficiency of the equal public good. We controlled for the overall efficiency across both public goods by including, for each condition, one trial with a small overall efficiency, one trial with an medium overall efficiency, and one trial with a high overall efficiency. Specifically, the unequal public good had lower efficiency than the equal public good (i.e., equal public good multiplier = 1.7 versus unequal public good multiplier = 1.5; 1.5 versus 1.3; and 1.3 versus 1.1), equal efficiency to the equal public good (i.e., equal public good multiplier = 1.7 versus unequal public good multiplier = 1.7; 1.5 versus 1.5, and 1.3 versus 1.3), or higher efficiency than the equal public good (i.e., equal public good 1.7 versus unequal public good 1.9; 1.5 versus 1.7; and 1.3 versus 1.5).

Participants were assigned to be low, intermediate, or high beneficiary from the unequal public good. As the reader may take from Table S1, the exact return rates provided subtle yet clear differences in unequal public good returns between group members, that further allowed us to isolate the effects of *relative* (dis-)advantage compared to the other group members from the *absolute* differences between equal- and unequal public good returns. For instance, in trial 5, the *low* beneficiary would receive the same return per MU from the equal- and unequal returns public good, but they would nonetheless receive comparatively less than their group members. Conversely, in trial 8, the *high* beneficiary would receive the same return per MU from the equal- and unequal returns public good, but would nonetheless receive comparatively more than the other group members.

In Experiment 1, all participants were assigned their beneficiary position at random. In Experiments 2 and 3, participants were informed that they were assigned to their beneficiary position either at random or based on an effort slider task (similar to^1^). Participants in the effort condition completed the effort slider task a few days before, at the first time point (Experiment 2), or right before the multiple-public goods game (Experiment 3). Since this random vs. effort manipulation did not yield significant differences in results, as reported in Hoenig and colleagues^2^, we averaged across these groups of participants.

**Table S1.** Individual returns per public good (PG) for each monetary unit contributed by

a group member, as presented to the participants on the decision screens.

| Block | Trial | Overall  Efficiency | Low Beneficiary | | Intermediate Beneficiary | | High Beneficiary | |
| --- | --- | --- | --- | --- | --- | --- | --- | --- |
|  |  |  | Equal PG | Unequal PG | Equal PG | Unequal PG | Equal PG | Unequal PG |
| Equal PG = Unequal PG | 1 | Low (1.3) | 0.43 | 0.36 | 0.43 | 0.43 | 0.43 | 0.50 |
|  | 2 | Medium (1.5) | 0.50 | 0.43 | 0.50 | 0.50 | 0.50 | 0.56 |
|  | 3 | High (1.7) | 0.56 | 0.50 | 0.56 | 0.56 | 0.56 | 0.63 |
| Equal PG < Unequal PG | 4 | Low (1.4) | 0.43 | 0.43 | 0.43 | 0.50 | 0.43 | 0.56 |
|  | 5 | Medium (1.6) | 0.50 | 0.50 | 0.50 | 0.56 | 0.50 | 0.63 |
|  | 6 | High (1.8) | 0.56 | 0.56 | 0.56 | 0.63 | 0.56 | 0.70 |
| Equal PG > Unequal PG | 7 | Low (1.2) | 0.43 | 0.30 | 0.43 | 0.36 | 0.43 | 0.43 |
|  | 8 | Medium (1.4) | 0.50 | 0.36 | 0.50 | 0.43 | 0.50 | 0.50 |
|  | 9 | High (1.6) | 0.56 | 0.43 | 0.56 | 0.50 | 0.56 | 0.56 |

*Note.* The order of conditions, as well as the order of trials within conditions, was randomized.

## 1.2 Measures

### 1.2.1 Ideology

Political Ideology was measured on a 7-point Likert scale, asking “On a left-right political spectrum, how would you describe your political orientation?” (UK) or “On a left-right political spectrum, how left or right are your political views?” (US), and ranging from “extreme left” to “extreme right”. We also measured ideology on a 7-point Likert scale, asking “On the following scale, how progressive or conservative are your political views?” (UK) or “On the following scale, how would you describe your political orientation?” (US) and ranging from “very progressive” (UK) or “very liberal” (US) to “very conservative”. Lastly, we measured economic ideology on a 7-point Likert scale, asking “On the following scale, how social or [neo-]liberal are your views on economic matters”? and ranging from “very social” to “very [neo-]liberal” (UK, [US]). See the distribution of political ideology in our sample in Figure S1.


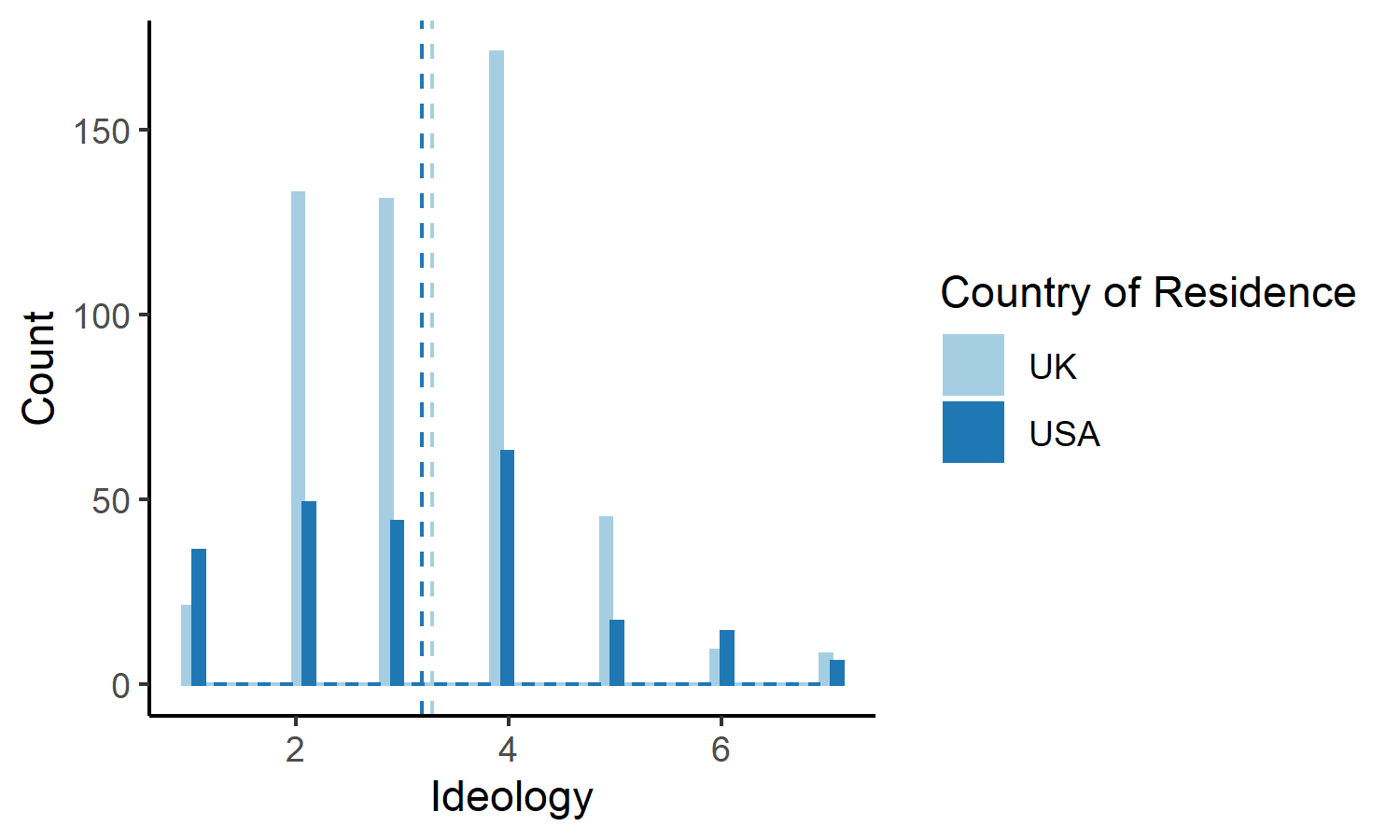


**Figure S1: Distribution of Ideology in our Sample**. The non-centered measure of political ideology, separated per country of Residence (UK versus USA). The dashed lines indicate the mean in the respective group.

### 1.2.2 Comprehension Questions

Participants had to respond correctly to the following comprehension questions before they could proceed to the multiple-public goods game. All questions were presented in randomized order. They were instructed as follows: “Please answer the few following questions to make sure that you understood the task correctly.”

1. The decisions by the other two members in my group will be made by other participants. (true/false)

2. The decisions of the other two participants will not influence my payment from this study. (true/false)

3. Sometimes, some members will earn more from a pool than others. (true/false)

4. The number by which contributions to a pool are multiplied will change across rounds. (true/false)

5. Who earns how much from an unequal pool... (will be known/will be unknown)

6. My decisions will influence my own payment and that of other participants. (true/false)

7. Imagine one of the pools in the task is equal and has a multiplier of 1.5 (return of 0.50 MU per member). How much would each member earn from this pool alone **if two contributed each 3 MUs and one contributed 4 MUs to this pool**? (3 MUs/4MUs/5MUs).

## 1.3 Statistical Analyses

Contributions are always averaged across the three levels, as across-public good efficiency did not play a role except for higher overall cooperation (for more information, see Hoenig and colleagues^2^). We include gender (female, male, and ‘other’ combining non-binary and self-defined gender) as control variable in all models.

## 1.4 Results

### 1.4.1 Cooperation

**Table S2.** Model predicting cooperation in the multiple-public goods game using the main

effects of Ideology (centered) and Public Good, as well as covariates for

Condition, Position, and Gender. Marginal R^2^ is 0.04 and conditional R^2^ is 0.20.

Marginal R^2^ describes the variance explained by all fixed effects, whereas

conditional R^2^ estimates the variance explained by all combined fixed effects

and random effects in the model. ‘PG’ represents the Public Good that

participants contributed to.

| Fixed Effect | β | Lower-95 | Higher-95 | *p*-value |
| --- | --- | --- | --- | --- |
| (Intercept) | 3.11 | 2.89 | 3.33 | <.001 |
| Ideology (centered) | -0.07 | -0.14 | 0.00 | .061 |
| PG (unequal PG) | -0.96 | -1.05 | -0.88 | <.001 |
| Condition (equal PG > unequal PG) | -0.24 | -0.34 | -0.13 | <.001 |
| Condition (equal PG = unequal PG) | -0.17 | -0.28 | -0.07 | .002 |
| Gender (male) | -0.03 | -0.23 | 0.16 | .747 |
| Gender (other) | 1.02 | 0.31 | 1.73 | .005 |
| Position (MB) | 0.24 | 0.01 | 0.47 | .044 |
| Position (HB) | 0.45 | 0.22 | 0.68 | <.001 |

**Table S3.** Model predicting cooperation in the multiple-public goods game using the two-

way interaction of Public Good and Condition, including the main effect of

Ideology, and Position and Gender as covariates. Marginal R^2^ is 0.12 and

conditional R^2^ is 0.29. ‘PG’ represents the Public Good that participants

contributed to.

| Fixed Effect | β | Lower-95 | Higher-95 | *p*-value |
| --- | --- | --- | --- | --- |
| (Intercept) | 2.00 | 1.77 | 2.22 | <.001 |
| Ideology (centered) | -0.07 | -0.14 | 0.00 | .061 |
| PG (unequal PG) | 1.26 | 1.11 | 1.40 | <.001 |
| Condition (equal PG > unequal PG) | 1.79 | 1.65 | 1.94 | <.001 |
| Condition (equal PG = unequal PG) | 1.12 | 0.98 | 1.27 | <.001 |
| Gender (male) | -0.03 | -0.23 | 0.16 | .747 |
| Gender (other) | 1.02 | 0.31 | 1.73 | .005 |
| Position (MB) | 0.24 | 0.01 | 0.47 | .044 |
| Position (HB) | 0.45 | 0.22 | 0.68 | <.001 |
| PG (unequal PG) * Condition  (equal PG > unequal PG) | -4.06 | -4.27 | -3.86 | <.001 |
| PG (unequal PG) * Condition  (equal PG = unequal PG) | -2.59 | -2.80 | -2.39 | <.001 |

**Table S4.** Model predicting cooperation in the multiple-public goods game using the two-

way interaction of Ideology and Public Good, including Condition, Position, and

Gender as covariates. Marginal R^2^ is 0.04 and conditional R^2^ is 0.20. ‘PG’

represents the Public Good that participants contributed to.

| Fixed Effect | β | Lower-95 | Higher-95 | *p*-value |
| --- | --- | --- | --- | --- |
| (Intercept) | 3.11 | 2.89 | 3.33 | <.001 |
| Ideology (centered) | -0.17 | -0.25 | -0.09 | <.001 |
| PG (unequal PG) | -0.96 | -1.05 | -0.88 | <.001 |
| Condition (equal PG > unequal PG) | -0.24 | -0.34 | -0.13 | <.001 |
| Condition (equal PG = unequal PG) | -0.17 | -0.28 | -0.07 | .002 |
| Gender (male) | -0.03 | -0.23 | 0.16 | .747 |
| Gender (other) | 1.02 | 0.31 | 1.73 | .005 |
| Position (MB) | 0.24 | 0.01 | 0.47 | .044 |
| Position (HB) | 0.45 | 0.22 | 0.68 | <.001 |
| Ideology (centered) * PG (unequal PG) | 0.20 | 0.13 | 0.26 | <.001 |

**Table S5.** Model predicting cooperation in the multiple-public goods game, using the three-way interaction of Ideology (centered), Public Good, and Condition, and

including Position and Gender as covariates. Marginal R^2^ is 0.12 and conditional

R^2^ is 0.30. ‘PG’ represents the Public Good that participants contributed to.

| Fixed Effect | β | Lower-95 | Higher-95 | *p*-value |
| --- | --- | --- | --- | --- |
| (Intercept) | 2.00 | 1.77 | 2.22 | <.001 |
| Ideology (centered) | -0.05 | -0.15 | 0.05 | .317 |
| PG (unequal PG) | 1.26 | 1.11 | 1.40 | <.001 |
| Condition (equal PG > unequal PG) | 1.79 | 1.65 | 1.94 | <.001 |
| Condition (equal PG = unequal PG) | 1.12 | 0.98 | 1.27 | <.001 |
| Gender (male) | -0.03 | -0.23 | 0.16 | .747 |
| Gender (other) | 1.02 | 0.31 | 1.73 | .005 |
| Position (MB) | 0.24 | 0.01 | 0.47 | .044 |
| Position (HB) | 0.45 | 0.22 | 0.68 | <.001 |
| Ideology (centered)*PG (unequal PG) | -0.05 | -0.15 | 0.06 | .410 |
| Ideology (centered)*Condition  (equal PG > unequal PG) | -0.22 | -0.33 | -0.11 | <.001 |
| Ideology (centered)*Condition  (equal PG = unequal PG) | -0.13 | -0.24 | -0.02 | .017 |
| PG (unequal PG)*Condition  (equal PG > unequal PG) | -4.06 | -4.26 | -3.86 | <.001 |
| PG (unequal PG)*Condition  (equal PG = unequal PG) | -2.59 | -2.79 | -2.39 | <.001 |
| Ideology (centered)*PG (unequal PG)* Condition (equal PG > unequal PG) | 0.43 | 0.28 | 0.59 | <.001 |
| Ideology (centered)*PG (unequal PG)* Condition (equal PG = unequal PG) | 0.30 | 0.14 | 0.45 | <.001 |

**Figure S2: Johnson-Neyman Analysis of the Interaction of Ideology and Public Good**. Johnson-Neyman intervals indicate where simple slopes are significant in the interactive effect of political ideology and public good on cooperation **(A)** and trust **(B)** in the multiple-public goods game, as well as descriptive expectations in the norm study **(C)**.

**A B**

**
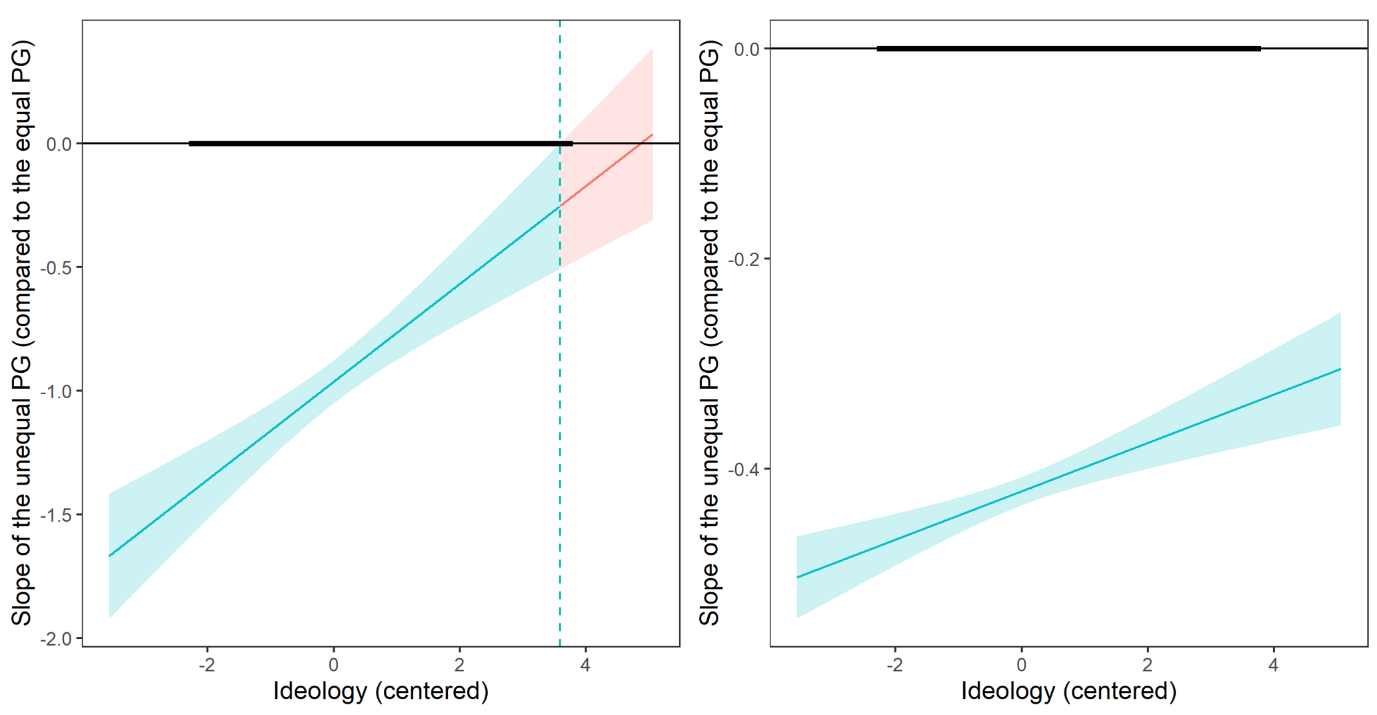
**

**C**

**
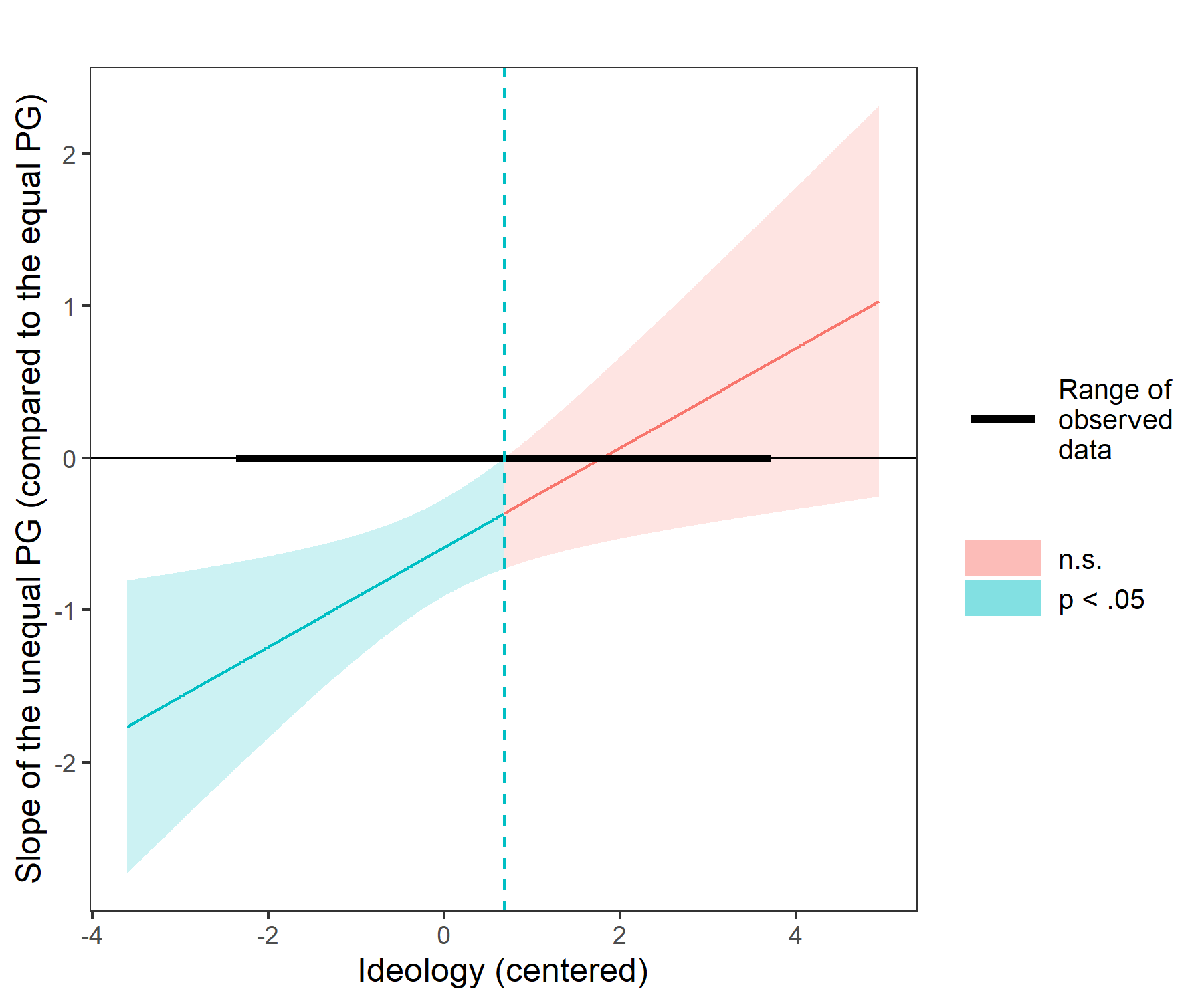
**

**Figure S3: Ideology predicts cooperation and trust**. Contributions to the equal and unequal public goods by self-identified left-leaning and right-leaning individuals when the efficiency of the unequal public good relative to the equal public good is higher, equal or lower **(A)**. Left-leaning and right-leaning individuals’ trust, measured as expectations of others’ contributions to the equal and unequal public good, when the efficiency of the unequal public good relative to the equal public good is higher, equal or lower **(B)**.

**A**

**
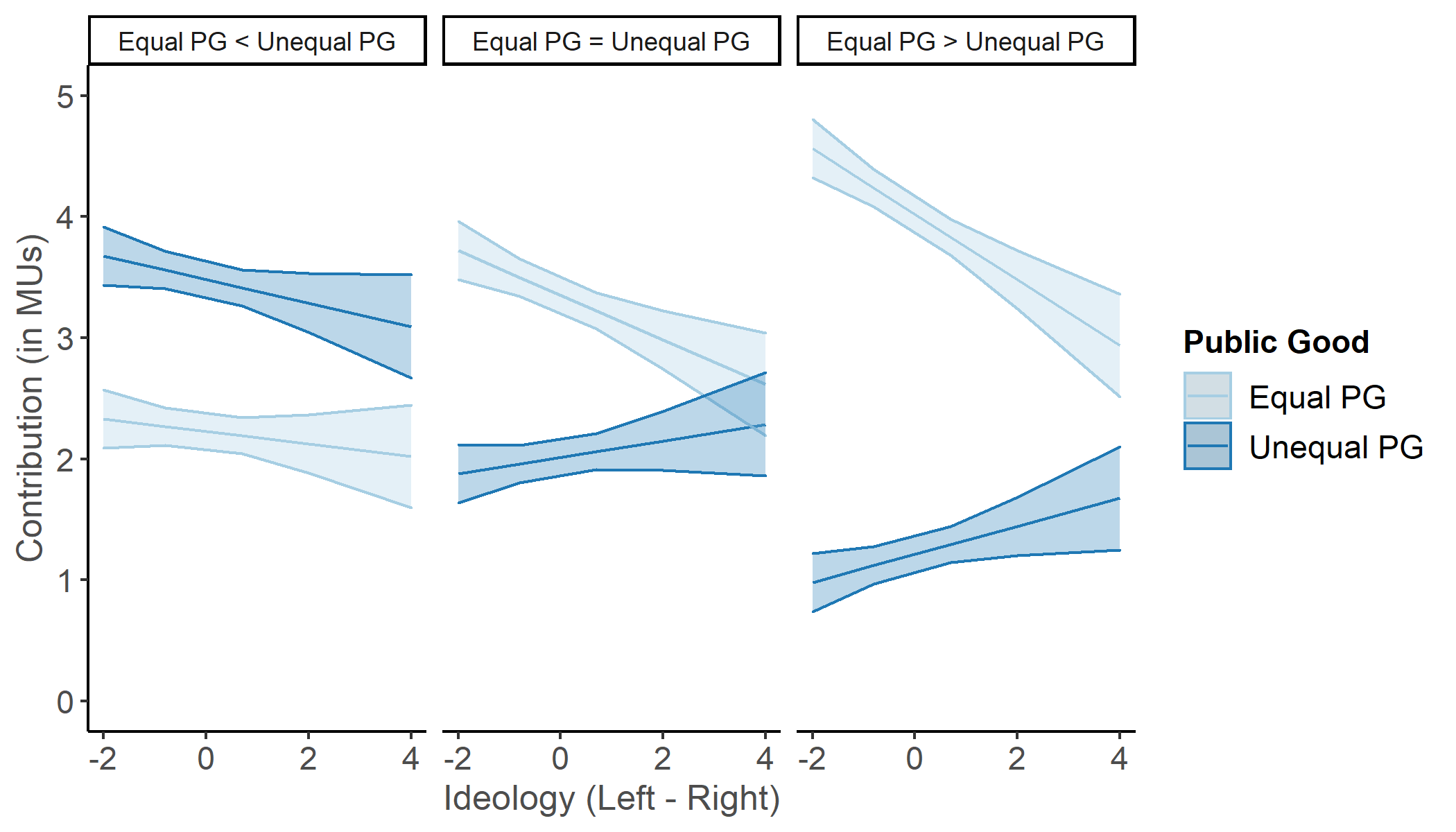
**

**B**

**
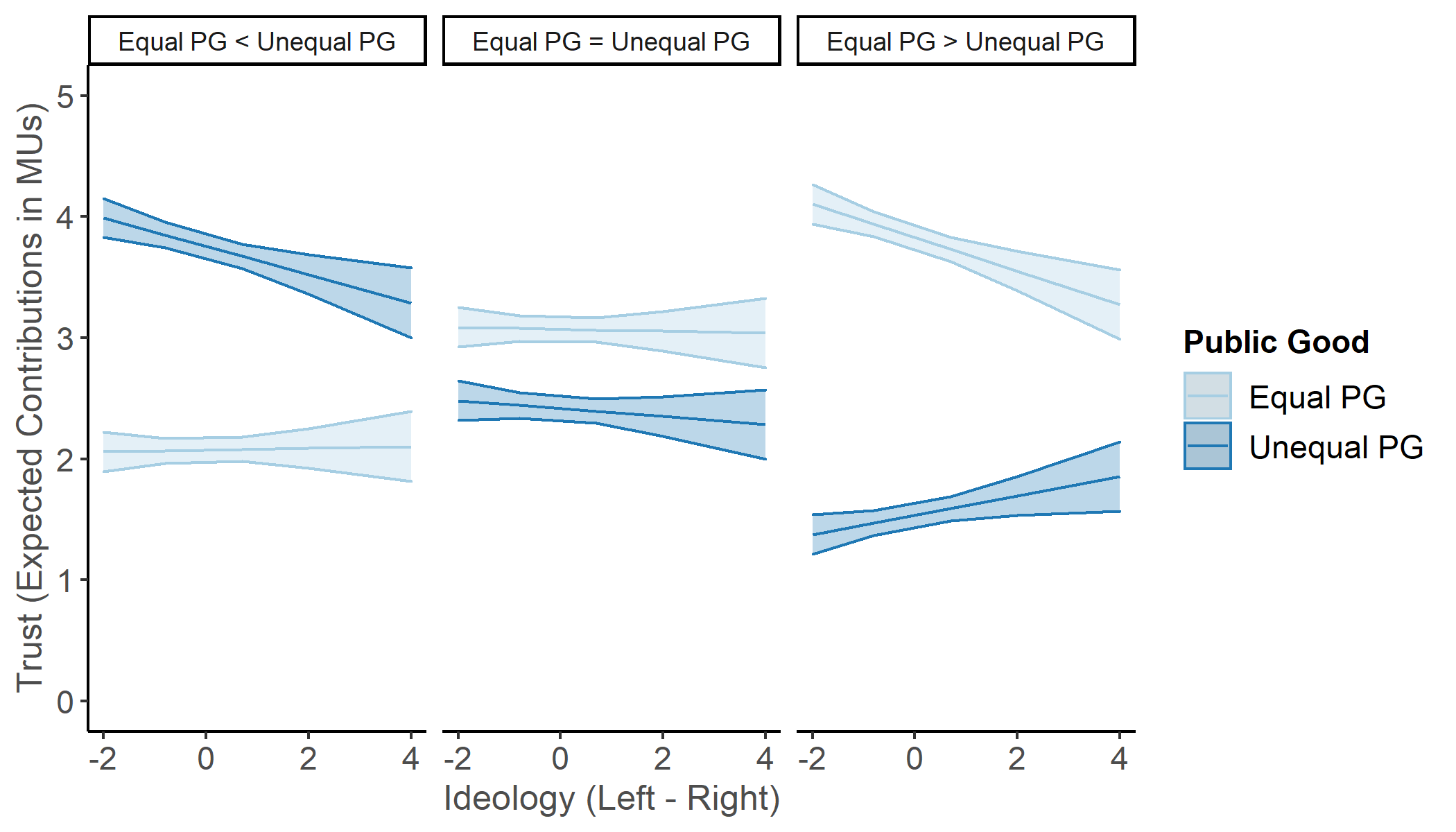
**

### 1.4.2 Trust

**Table S6.** Model predicting trust in the multiple-public goods game using only main effects

of Ideology (centered) and Public Good, including Condition, Position, and

Gender as covariates. Marginal R^2^ is 0.01 and conditional R^2^ is 0.21. ‘PG’

represents the Public Good that participants contributed to.

| Fixed Effect | β | Lower-95 | Higher-95 | *p*-value |
| --- | --- | --- | --- | --- |
| (Intercept) | 3.19 | 3.00 | 3.38 | <.001 |
| Ideology (centered) | -0.03 | -0.10 | 0.03 | .314 |
| PG (unequal PG) | -0.42 | -0.44 | -0.41 | <.001 |
| Condition (equal PG = unequal PG) | -0.17 | -0.19 | -0.16 | <.001 |
| Condition (equal PG > unequal PG) | -0.23 | -0.25 | -0.22 | <.001 |
| Gender (male) | -0.06 | -0.24 | 0.12 | .483 |
| Gender (other) | 0.88 | 0.22 | 1.53 | .009 |
| Position (IB) | -0.03 | -0.24 | 0.18 | .784 |
| Position (HB) | -0.10 | -0.31 | 0.11 | .358 |

**Table S7.** Model predicting trust in the multiple-public goods game using the two-way

interaction of Public Good and Condition, including Ideology (centered),

Position, and Gender as covariates. Marginal R^2^ is 0.01 and conditional R^2^ is

0.21. ‘PG’ represents the Public Good that participants contributed to.

| Fixed Effect | β | Lower-95 | Higher-95 | *p*-value |
| --- | --- | --- | --- | --- |
| (Intercept) | 2.14 | 1.95 | 2.33 | <.001 |
| Ideology (centered) | -0.03 | -0.10 | 0.03 | .314 |
| PG (Unequal PG) | 1.68 | 1.66 | 1.70 | <.001 |
| Condition (A=B) | 1.00 | 0.97 | 1.02 | <.001 |
| Condition (A>B) | 1.75 | 1.73 | 1.77 | <.001 |
| Gender (male) | -0.06 | -0.24 | 0.12 | .483 |
| Gender (other) | 0.88 | 0.22 | 1.53 | .009 |
| Position (IB) | -0.03 | -0.24 | 0.18 | .784 |
| Position (HB) | -0.10 | -0.31 | 0.11 | .358 |
| Ideology (centered) * Condition (A=B) | -2.34 | -2.37 | -2.31 | <.001 |
| Ideology (centered) * Condition (A>B) | -3.97 | -4.00 | -3.94 | <.001 |

**Table S8.** Model predicting trust in the multiple-public goods game using the two-way

interaction of Ideology (centered) and Public Good, including Condition,

Position, and Gender as covariates. Marginal R^2^ is 0.01 and conditional R^2^ is

0.21. ‘PG’ represents the Public Good that participants contributed to.

| Fixed Effect | β | Lower-95 | Higher-95 | *p*-value |
| --- | --- | --- | --- | --- |
| (Intercept) | 3.19 | 3.00 | 3.38 | <.001 |
| Ideology (centered) | -0.05 | -0.11 | 0.03 | .181 |
| PG (unequal PG) | -0.42 | -0.44 | -0.41 | <.001 |
| Condition (equal PG = unequal PG) | -0.17 | -0.19 | -0.16 | <.001 |
| Condition (equal PG > unequal PG) | -0.23 | -0.25 | -0.22 | <.001 |
| Gender (male) | -0.06 | -0.24 | 0.12 | .483 |
| Gender (other) | 0.88 | 0.22 | 1.53 | .009 |
| Position (IB) | -0.03 | -0.24 | 0.18 | .784 |
| Position (HB) | -0.10 | -0.31 | 0.11 | .358 |
| Ideology (centered) * PG (unequal PG) | 0.02 | 0.01 | 0.03 | <.001 |

**Table S9.** Model predicting trust in the multiple-public goods game using the three-way

interaction of Ideology (centered), Public Good, and Condition, including

Position and Gender as covariates. Marginal R^2^ is 0.10 and conditional R^2^ is

0.30. ‘PG’ represents the Public Good that participants contributed to.

| Fixed Effect | β | Lower-95 | Higher-95 | *p*-value |
| --- | --- | --- | --- | --- |
| (Intercept) | 2.14 | 1.95 | 2.33 | <.001 |
| Ideology (centered) | 0.01 | -0.06 | 0.08 | .836 |
| PG (Unequal PG) | 1.68 | 1.66 | 1.70 | <.001 |
| Condition (A=B) | 1.00 | 0.97 | 1.02 | <.001 |
| Condition (A>B) | 1.75 | 1.73 | 1.77 | <.001 |
| Gender (male) | -0.06 | -0.24 | 0.12 | .483 |
| Gender (other) | 0.88 | 0.22 | 1.53 | .009 |
| Position (IB) | -0.03 | -0.24 | 0.18 | .784 |
| Position (HB) | -0.10 | -0.31 | 0.11 | .358 |
| Ideology (centered)* PG (Unequal PG) | -0.12 | -0.14 | -0.11 | <.001 |
| Ideology (centered)*Condition (A=B) | -0.02 | -0.03 | 0.00 | .074 |
| Ideology (centered)*Condition (A>B) | -0.14 | -0.16 | -0.13 | <.001 |
| PG (Unequal PG)*Condition (A=B) | -2.34 | -2.37 | -2.31 | <.001 |
| PG (Unequal PG)*Condition (A>B) | -3.97 | -4.00 | -3.94 | <.001 |
| Ideology (centered)* PG (Unequal PG)* Condition (A=B) | 0.10 | 0.08 | 0.12 | <.001 |
| Ideology (centered)* PG (Unequal PG))* Condition (A>B) | 0.34 | 0.32 | 0.37 | <.001 |

### 1.4.2.1 Mediation of Ideology and Cooperation through Trust

Albeit trust about others’ cooperation in the multiple-public goods game was similarly shaped by an interaction of ideology and public good as was cooperative behavior itself, trust did not significantly mediate the relationship between ideology and cooperation.

To investigate this mediation, we performed two separate analyses for equal public good cooperation (Tables S10 and S11) and unequal public good cooperation (Tables S12 and S13), respectively, using the *mediation* package in R. For both equal and unequal public good cooperation, the first mediation model predicting trust failed to produce a significant main effect of ideology on trust (see Tables S10 and S12).

**Table S10.** Mediation model 1. Model predicting trust of equal public good contributions

using the main effects of Ideology (centered), Condition, own Position, and

Gender as covariates.

| Fixed Effect | β | Lower-95 | Higher-95 | *p*-value |
| --- | --- | --- | --- | --- |
| (Intercept) | 1.81 | 1.56 | 2.07 | <.001 |
| Ideology (centered) | -.04 | -0.13 | 0.05 | .425 |
| Condition (equal PG = unequal PG) | 1.00 | 0.95 | 1.05 | <.001 |
| Condition (equal PG > unequal PG) | 1.75 | 1.70 | 1.80 | <.001 |
| Gender (male) | -0.11 | -0.35 | 0.12 | .353 |
| Gender (other) | 1.18 | 0.31 | 2.05 | .008 |
| Position (IB) | 0.31 | 0.03 | 0.59 | .033 |
| Position (HB) | 0.60 | 0.32 | 0.89 | <.001 |

**Table S11.** Mediation model 2. Model predicting cooperation on the equal public good using

the main effects of Trust, Ideology (centered), Condition, own Position, and

Gender as covariates.

| Fixed Effect | β | Lower-95 | Higher-95 | *p*-value |
| --- | --- | --- | --- | --- |
| (Intercept) | 1.94 | 1.66 | 2.21 | <.001 |
| Trust | 0.24 | 0.23 | 0.25 | <.001 |
| Ideology (centered) | -0.16 | -0.26 | -0.07 | <.001 |
| Condition (equal PG = unequal PG) | 0.88 | 0.84 | 0.93 | <.001 |
| Condition (equal PG > unequal PG) | 1.37 | 1.32 | 1.42 | <.001 |
| Gender (male) | 0.04 | -0.22 | 0.30 | .760 |
| Gender (other) | 0.92 | -0.02 | 1.86 | .057 |
| Position (IB) | -0.14 | -0.44 | 0.17 | .379 |
| Position (HB) | -0.61 | -0.92 | -0.30 | <.001 |

**Table S12.** Mediation model 1. Model predicting trust of unequal public good contributions

using the main effects of Ideology (centered), Condition, own Position, and Gender as covariates.

| Fixed Effect | β | Lower-95 | Higher-95 | *p*-value |
| --- | --- | --- | --- | --- |
| (Intercept) | 4.14 | 3.94 | 4.35 | <.001 |
| Ideology (centered) | -0.03 | -0.11 | 0.04 | .376 |
| Condition (equal PG = unequal PG) | -1.34 | -1.39 | -1.29 | <.001 |
| Condition (equal PG > unequal PG) | -2.22 | -2.27 | -2.17 | <.001 |
| Gender (male) | -0.02 | -0.21 | 0.18 | .874 |
| Gender (other) | 0.58 | -0.13 | 1.28 | .110 |
| Position (IB) | -0.37 | -0.60 | -0.14 | .002 |
| Position (HB) | -0.80 | -1.03 | -0.57 | <.001 |

**Table S13.** Mediation model 2. Model predicting cooperation on the unequal public good

using the main effects of Trust, Ideology (centered), Condition, own Position,

and Gender as covariates.

| Fixed Effect | β | Lower-95 | Higher-95 | *p*-value |
| --- | --- | --- | --- | --- |
| (Intercept) | 2.00 | 1.79 | 2.22 | <.001 |
| Trust | 0.21 | 0.20 | 0.22 | <.001 |
| Ideology (centered) | 0.04 | -0.03 | 0.11 | .294 |
| Condition (equal PG = unequal PG) | -1.19 | -1.23 | -1.14 | <.001 |
| Condition (equal PG > unequal PG) | -1.80 | -1.85 | -1.75 | <.001 |
| Gender (male) | -0.07 | -0.27 | 0.12 | .463 |
| Gender (other) | 0.72 | 0.00 | 1.44 | .051 |
| Position (IB) | 0.62 | 0.38 | 0.85 | <.001 |
| Position (HB) | 1.53 | 1.30 | 1.77 | <.001 |

### 1.4.3 The role of Social Value Orientation

Social value orientation (SVO) has been shown to be an essential predictor of cooperation in public good provision across a myriad of paradigms and, at times, to be related to political ideology. We measured SVO using the SVO slider measure^3^ either at the first time point, thus several days before the economic game (Experiments 1 and 2), or after the economic game in case all tasks were completed at one time point (Experiment 3).

Also in our sample, the SVO angle correlated significantly with political ideology (Pearson correlation: *r*(13444) = -.04, 95% CI = [-0.06, -0.03], *p* < .001). More specifically, the more left leaning a participant, the more prosocial the participant was in the SVO slider measure. We further tested if ideology has a unique influence on cooperation in our paradigm by entering both political ideology and SVO alone and in interaction with the (un)equal public good, controlling for Position, Condition, and Gender (see Tables S14-16).

Predicting overall cooperation, in comparison to the model excluding SVO, political ideology remains non-significant (but trending), while SVO shows that more prosocial individuals cooperate more overall than do more pro-self-oriented individuals (see main effects in Table S14). However, self-identified left-leaning individuals cooperated significantly more on the equal public good than on the unequal public good and right-leaning individuals do not differentiate between the two public goods, also when controlling for SVO (Table S15). That SVO did not act as moderator for ideology and the (un)equal public good in predicting cooperation is shown by the non-significant three-way interaction (Table S16).

**Table S14.** Model predicting cooperation in the multiple-public goods game using only

main effects, including Position and Gender as covariates. Marginal R^2^ is 0.06

and conditional R^2^ is 0.20. ‘PG’ represents the Public Good that participants

contributed to and ‘SVO’ represents Social Value Orientation.

| Fixed Effect | β | Lower-95 | Higher-95 | *p*-value |
| --- | --- | --- | --- | --- |
| (Intercept) | 1.88 | 1.56 | 2.21 | <.001 |
| Ideology (centered) | -0.06 | -0.13 | 0.01 | .097 |
| SVO | 0.04 | 0.03 | 0.05 | <.001 |
| PG (unequal PG) | -0.96 | -1.05 | -0.88 | <.001 |
| Condition (equal PG > unequal PG) | -0.24 | -0.34 | -0.13 | <.001 |
| Condition (equal PG = unequal PG) | -0.17 | -0.28 | -0.07 | .002 |
| Gender (male) | -0.06 | -0.24 | 0.13 | .549 |
| Gender (other) | 0.93 | 0.25 | 1.60 | .007 |
| Position (MB) | 0.16 | -0.06 | 0.38 | .159 |
| Position (HB) | 0.40 | 0.18 | 0.61 | <.001 |

**Table S15.** Model predicting cooperation in the multiple-public goods game using the two-

way interaction of Ideology (centered) and Public Good, including SVO,

Condition, Position and Gender as covariates. Marginal R^2^ is 0.06 and

conditional R^2^ is 0.20. ‘PG’ represents the Public Good that participants

contributed to and ‘SVO’ represents Social Value Orientation.

| Fixed Effect | β | Lower-95 | Higher-95 | *p*-value |
| --- | --- | --- | --- | --- |
| (Intercept) | 1.88 | 1.56 | 2.21 | <.001 |
| Ideology (centered) | -0.16 | -0.23 | -0.08 | <.001 |
| SVO | 0.04 | 0.03 | 0.05 | <.001 |
| PG (unequal PG) | -0.96 | -1.05 | -0.88 | <.001 |
| Condition (equal PG > unequal PG) | -0.24 | -0.34 | -0.13 | <.001 |
| Condition (equal PG = unequal PG) | -0.17 | -0.28 | -0.07 | .002 |
| Gender (male) | -0.06 | -0.24 | 0.13 | .549 |
| Gender (other) | 0.93 | 0.25 | 1.60 | .007 |
| Position (MB) | 0.16 | -0.06 | 0.38 | .159 |
| Position (HB) | 0.40 | 0.18 | 0.61 | <.001 |
| Ideology (centered) * PG (unequal PG) | 0.20 | 0.13 | 0.26 | <.001 |

**Table S16.** Model predicting cooperation in the multiple-public goods game using the three-

way interaction of Ideology (centered), Public Good, and SVO, including

Condition, Position, and Gender as covariates. Marginal R^2^ is 0.07 and

conditional R^2^ is 0.21. ‘PG’ represents the Public Good that participants

contributed to and ‘SVO’ represents Social Value Orientation.

| Fixed Effect | β | Lower-95 | Higher-95 | *p*-value |
| --- | --- | --- | --- | --- |
| (Intercept) | 1.46 | 1.56 | 2.21 | <.001 |
| Ideology (centered) | -0.28 | -0.53 | -0.03 | .026 |
| SVO | 0.06 | 0.05 | 0.07 | <.001 |
| PG (unequal PG) | -0.12 | -0.39 | 0.14 | .365 |
| Condition (equal PG > unequal PG) | -0.24 | -0.34 | -0.13 | <.001 |
| Condition (equal PG = unequal PG) | -0.17 | -0.28 | -0.07 | .002 |
| Gender (male) | -0.06 | -0.24 | 0.13 | .536 |
| Gender (other) | 0.93 | 0.26 | 1.61 | .007 |
| Position (MB) | 0.16 | -0.06 | 0.38 | .155 |
| Position (HB) | 0.40 | 0.18 | 0.62 | <.001 |
| Ideology (centered) * PG (unequal PG) | 0.36 | 0.14 | 0.57 | .001 |
| Ideology (centered) * SVO | 0.00 | 0.00 | 0.01 | .281 |
| PG (unequal PG) * SVO | -0.03 | -0.04 | -0.02 | <.001 |
| Ideology (centered) * PG * SVO | -0.01 | -0.01 | 0.00 | .108 |

# 2 Norm Study

## 2.1 Measures

### 2.1.1 Comprehension Questions

Participants had to respond correctly to the following comprehension questions before they could proceed to indicating normative and descriptive expectations. All questions were presented in randomized order. They were instructed that they could use a calculator if needed.

1. What were the returns from Project A if two group members contributed 3 MUs each, and one contributed 4 MUs to it?

(Each group member received 3 MUs./ Each group member received 5 MUs./ Each group member received a different amount.)

2. When members' investments in Project B totaled 18 MUs...

(Member 1 received 3 MUs, Member 2 received 6 MUs, and Member 3 received 9 MUs./

Member 1 received 2 MUs, Member 2 received 4 MUs, and Member 3 received 6 MUs./

Member 1 received 9 MUs, Member 2 received 6 MUs, and Member 3 received 3 MUs./

All group members received 6 MUs.)

## 2.2 Results

**Table S17.** Model predicting normative expectations and descriptive expectations using main effects, including the Position of the target and Gender as covariates.

Marginal R^2^ is 0.19 and 0.04 respectively, and conditional R^2^ is 0.19 and 0.09,

respectively. ‘PG’ represents the Public Good that participants contributed to.

| Fixed Effect | β | Lower-95 | Higher-95 | *p*-value |
| --- | --- | --- | --- | --- |
| **Normative Expectations** | | | | |
| (Intercept) | 4.63 | 4.26 | 4.99 | <.001 |
| Ideology (centered) | -0.10 | -0.23 | 0.03 | .148 |
| PG (unequal PG) | -2.02 | -2.35 | -1.70 | <.001 |
| Target Position (IB) | 0.17 | -0.23 | 0.57 | .400 |
| Target Position (HB) | 0.58 | 0.18 | 0.98 | .005 |
| Gender (male) | -0.31 | -0.64 | 0.02 | .068 |
| Gender (other) | 0.91 | -0.92 | 2.74 | .330 |
| **Descriptive Expectations** | | | | |
| (Intercept) | 3.11 | 2.73 | 3.50 | <.001 |
| Ideology (centered) | 0.01 | -0.14 | 0.16 | .936 |
| PG (unequal PG) | -0.59 | -0.91 | -0.27 | <.001 |
| Target Position (IB) | 0.43 | 0.03 | 0.82 | .033 |
| Target Position (HB) | 0.63 | 0.24 | 1.03 | .002 |
| Gender (male) | 0.21 | -0.16 | 0.59 | .266 |
| Gender (other) | 1.84 | -0.25 | 3.93 | .084 |

**Table S18.** Model predicting normative expectations and descriptive expectations using

the interaction of Ideology (centered) and Public Good, including the Position of

the target and Gender as covariates. Marginal R^2^ is 0.18 and 0.03 respectively,

and conditional R^2^ is 0.18 and 0.09, respectively. ‘PG’ represents the Public

Good that participants contributed to.

| Fixed Effect | β | Lower-95 | Higher-95 | *p*-value |
| --- | --- | --- | --- | --- |
| **Normative Expectations** | | | | |
| (Intercept) | 4.63 | 4.26 | 4.99 | <.001 |
| Ideology (centered) | -0.19 | -0.37 | -0.01 | .043 |
| PG (unequal PG) | -2.02 | -2.35 | -1.70 | <.001 |
| Target Position (IB) | 0.17 | -0.23 | 0.57 | .400 |
| Target Position (HB) | 0.58 | 0.18 | 0.98 | .004 |
| Gender (male) | -0.31 | -0.64 | 0.02 | .068 |
| Gender (other) | 0.91 | -0.92 | 2.74 | .330 |
| Ideology (centered)* PG (unequal PG) | 0.19 | -0.07 | 0.44 | .155 |
| **Descriptive Expectations** | | | | |
| (Intercept) | 3.11 | 2.73 | 3.49 | <.001 |
| Ideology (centered) | -0.16 | -0.35 | 0.04 | .115 |
| PG (unequal PG) | -0.59 | -0.91 | -0.27 | <.001 |
| Target Position (IB) | 0.43 | 0.04 | 0.82 | .032 |
| Target Position (HB) | 0.63 | 0.24 | 1.03 | .002 |
| Gender (male) | 0.21 | -0.16 | 0.59 | .266 |
| Gender (other) | 1.84 | -0.25 | 3.93 | .084 |
| Ideology (centered)* PG (unequal PG) | 0.33 | 0.08 | 0.58 | .011 |

## 2.3 Visuals of Norm Study

**Figure S4: Instructions to the measure of normative and descriptive expectations**. The instructions in **A-D** were followed by examples and comprehension questions, before proceeding to the decision slides in **E** for normative expectations and **F** for descriptive expectations.

**A**


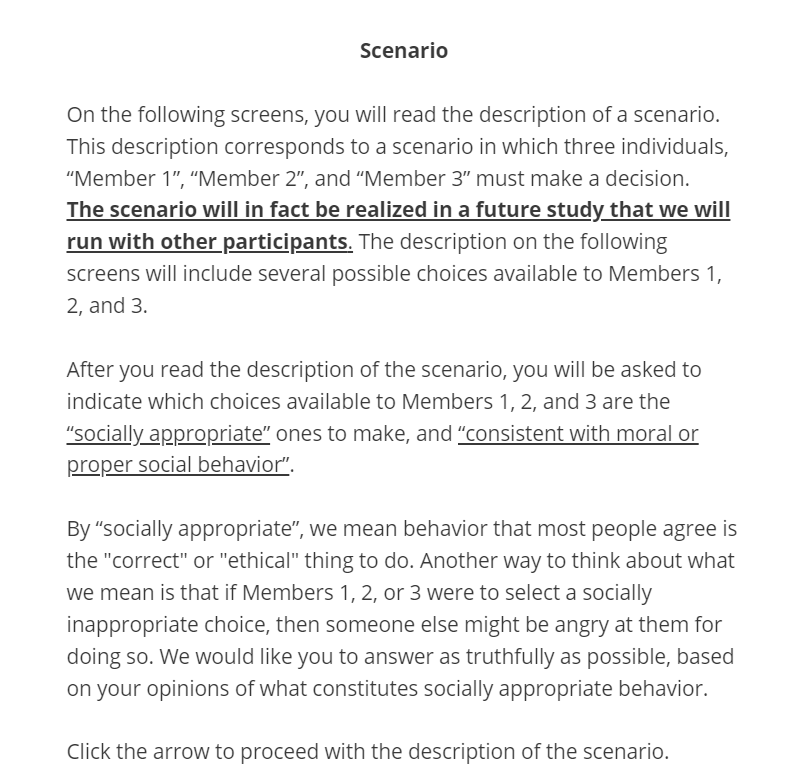


**B**


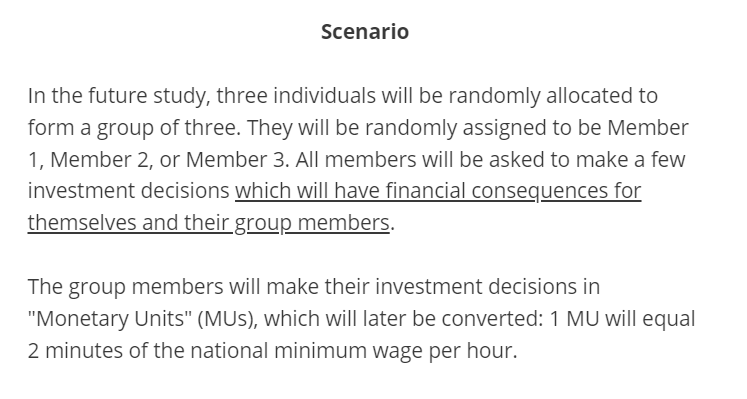


**C**


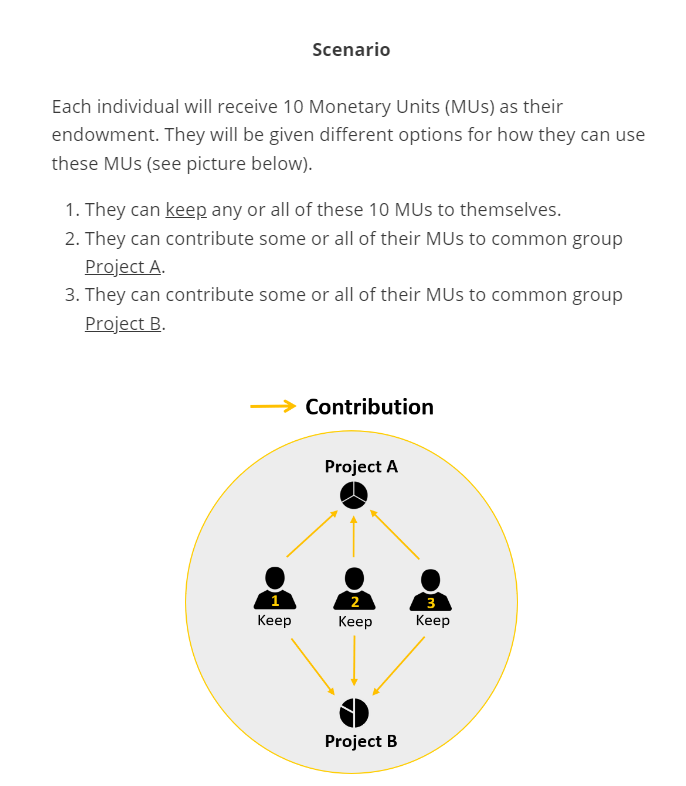


**D**


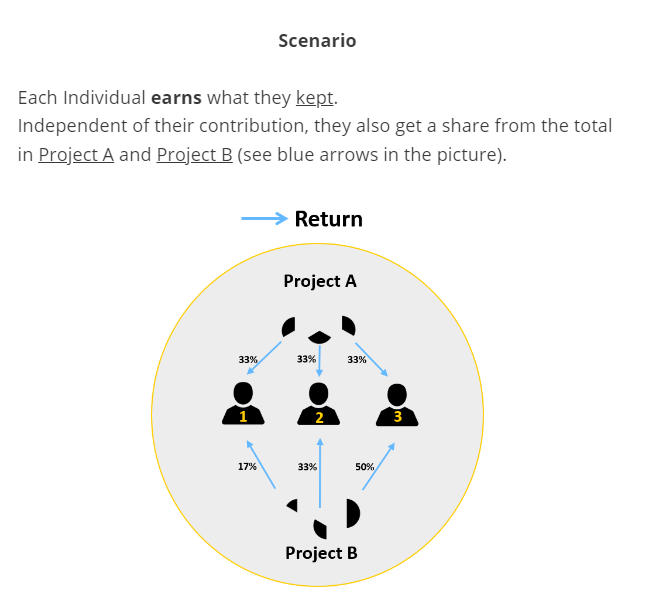


**E**


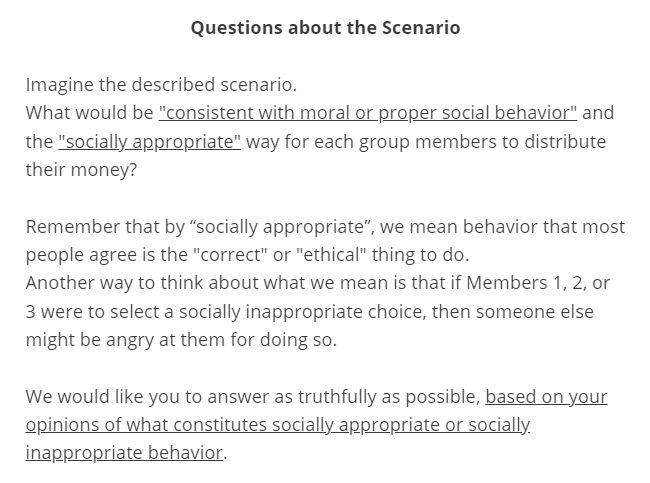


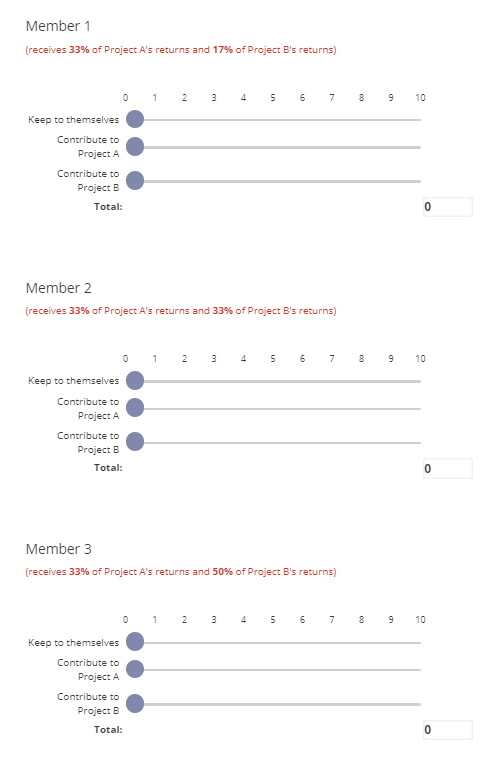


**F**


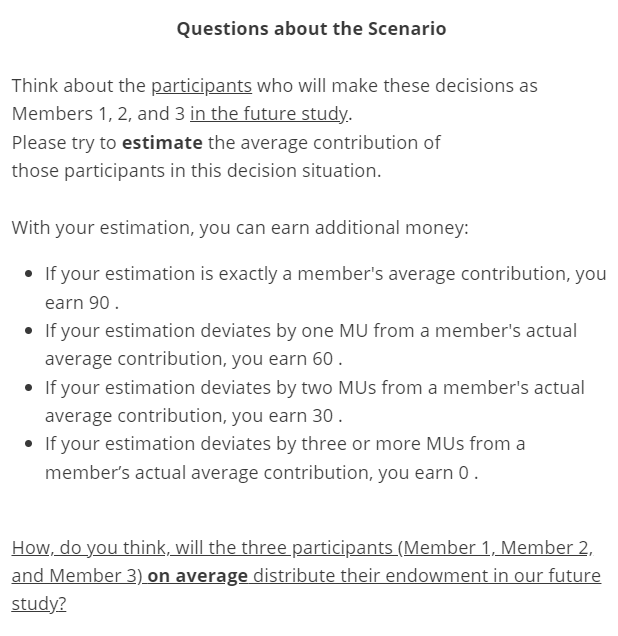


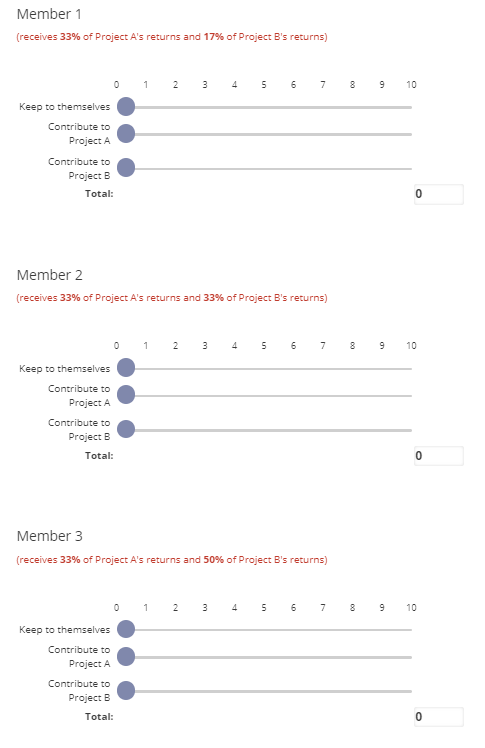


# 3 Main Analyses controlling for Country of Residence

## 3.1 Cooperation

**Table S19.** Model predicting cooperation in the multiple-public goods game using the main

effects of Ideology (centered) and Public Good, as well as covariates for

Condition, Position, Gender, and Country of Residence. Marginal R^2^ is 0.04 and

conditional R^2^ is 0.20. Marginal R^2^ describes the variance explained by all fixed

effects, whereas conditional R^2^ estimates the variance explained by all combined

fixed effects and random effects in the model. ‘PG’ represents the Public Good

that participants contributed to.

| Fixed Effect | β | Lower-95 | Higher-95 | *p*-value |
| --- | --- | --- | --- | --- |
| (Intercept) | 3.08 | 2.84 | 3.31 | <.001 |
| Ideology (centered) | -0.07 | -0.14 | 0.00 | .067 |
| PG (unequal PG) | -0.96 | -1.05 | -0.88 | <.001 |
| Condition (equal PG > unequal PG) | -0.24 | -0.34 | -0.13 | <.001 |
| Condition (equal PG = unequal PG) | -0.17 | -0.28 | -0.07 | .002 |
| Gender (male) | -0.02 | -0.22 | 0.18 | .845 |
| Gender (other) | 1.03 | 0.31 | 1.74 | .005 |
| Position (MB) | 0.24 | 0.01 | 0.48 | .040 |
| Position (HB) | 0.45 | 0.22 | 0.68 | <.001 |
| Residence | 0.07 | -0.14 | 0.28 | .508 |

**Table S20.** Model predicting cooperation in the multiple-public goods game using the two-

way interaction of Public Good and Condition, including the main effect of

Ideology, and Position, Gender, and Country of Residence as covariates.

Marginal R^2^ is 0.12 and conditional R^2^ is 0.29. ‘PG’ represents the Public Good

that participants contributed to.

| Fixed Effect | β | Lower-95 | Higher-95 | *p*-value |
| --- | --- | --- | --- | --- |
| (Intercept) | 1.97 | 1.72 | 2.21 | <.001 |
| Ideology (centered) | -0.07 | -0.14 | 0.00 | .067 |
| PG (unequal PG) | 1.26 | 1.11 | 1.40 | <.001 |
| Condition (equal PG > unequal PG) | 1.80 | 1.65 | 1.94 | <.001 |
| Condition (equal PG = unequal PG) | 1.13 | 0.98 | 1.27 | <.001 |
| Gender (male) | -0.02 | -0.22 | 0.18 | .845 |
| Gender (other) | 1.03 | 0.31 | 1.74 | .005 |
| Position (MB) | 0.24 | 0.01 | 0.48 | .040 |
| Position (HB) | 0.45 | 0.22 | 0.68 | <.001 |
| Residence | 0.07 | -0.14 | 0.28 | .508 |
| PG (unequal PG) * Condition  (equal PG > unequal PG) | -4.07 | -4.27 | -3.86 | <.001 |
| PG (unequal PG) * Condition  (equal PG = unequal PG) | -2.60 | -2.80 | -2.39 | <.001 |

**Table S21.** Model predicting cooperation in the multiple-public goods game using the two-

way interaction of Ideology and Public Good, including Condition, Position,

Gender, and Country of Residence as covariates. Marginal R^2^ is 0.04 and

conditional R^2^ is 0.20. ‘PG’ represents the Public Good that participants

contributed to.

| Fixed Effect | β | Lower-95 | Higher-95 | *p*-value |
| --- | --- | --- | --- | --- |
| (Intercept) | 3.08 | 2.84 | 3.31 | <.001 |
| Ideology (centered) | -0.17 | -0.25 | -0.09 | <.001 |
| PG (unequal PG) | -0.96 | -1.05 | -0.88 | <.001 |
| Condition (equal PG > unequal PG) | -0.24 | -0.34 | -0.13 | <.001 |
| Condition (equal PG = unequal PG) | -0.17 | -0.28 | -0.07 | .002 |
| Gender (male) | -0.02 | -0.22 | 0.18 | .845 |
| Gender (other) | 1.03 | 0.31 | 1.74 | .005 |
| Position (MB) | 0.24 | 0.01 | 0.48 | .040 |
| Position (HB) | 0.45 | 0.22 | 0.68 | <.001 |
| Residence | 0.07 | -0.14 | 0.28 | .508 |
| Ideology (centered) * PG (unequal PG) | 0.20 | 0.13 | 0.26 | <.001 |

**Table S22.** Model predicting cooperation in the multiple-public goods game, using the

three-way interaction of Ideology, Public Good, and Condition, and including

Position, Gender, and Country of Residence as covariates. Marginal R^2^ is 0.13

and conditional R^2^ is 0.30. ‘PG’ represents the Public Good that participants

contributed to.

| Fixed Effect | β | Lower-95 | Higher-95 | *p*-value |
| --- | --- | --- | --- | --- |
| (Intercept) | 1.97 | 1.72 | 2.21 | <.001 |
| Ideology (centered) | -0.05 | -0.15 | 0.05 | .331 |
| PG (unequal PG) | 1.26 | 1.11 | 1.40 | <.001 |
| Condition (equal PG > unequal PG) | 1.80 | 1.65 | 1.94 | <.001 |
| Condition (equal PG = unequal PG) | 1.13 | 0.98 | 1.27 | <.001 |
| Gender (male) | -0.02 | -0.22 | 0.18 | .845 |
| Gender (other) | 1.03 | 0.31 | 1.74 | .005 |
| Position (MB) | 0.24 | 0.01 | 0.48 | .040 |
| Position (HB) | 0.45 | 0.22 | 0.68 | <.001 |
| Residence | 0.07 | -0.14 | 0.28 | .508 |
| Ideology (centered)*PG (unequal PG) | -0.05 | -0.15 | 0.06 | .410 |
| Ideology (centered)*Condition  (equal PG > unequal PG) | -0.22 | -0.33 | -0.11 | <.001 |
| Ideology (centered)*Condition  (equal PG = unequal PG) | -0.13 | -0.24 | -0.02 | .017 |
| PG (unequal PG)*Condition  (equal PG > unequal PG) | -4.07 | -4.26 | -3.86 | <.001 |
| PG (unequal PG)*Condition  (equal PG = unequal PG) | -2.60 | -2.79 | -2.39 | <.001 |
| Ideology (centered)*PG (unequal PG)* Condition (equal PG > unequal PG) | 0.43 | 0.28 | 0.59 | <.001 |
| Ideology (centered)*PG (unequal PG)* Condition (equal PG = unequal PG) | 0.30 | 0.14 | 0.45 | <.001 |

## 3.2 Trust

**Table S23.** Model predicting trust in the multiple-public goods game using only main effects

of Ideology (centered) and Public Good, including Condition, Position, Gender,

and Country of Residence as covariates. Marginal R^2^ is 0.01 and conditional R^2^

is 0.21. ‘PG’ represents the Public Good that participants contributed to.

| Fixed Effect | β | Lower-95 | Higher-95 | *p*-value |
| --- | --- | --- | --- | --- |
| (Intercept) | 3.16 | 2.95 | 3.37 | <.001 |
| Ideology (centered) | -0.03 | -0.10 | 0.03 | .336 |
| PG (unequal PG) | -0.42 | -0.44 | -0.41 | <.001 |
| Condition (equal PG = unequal PG) | -0.17 | -0.19 | -0.16 | <.001 |
| Condition (equal PG > unequal PG) | -0.23 | -0.25 | -0.22 | <.001 |
| Gender (male) | -0.05 | -0.23 | 0.13 | .583 |
| Gender (other) | 0.88 | 0.23 | 1.54 | .009 |
| Position (IB) | -0.03 | -0.24 | 0.19 | .820 |
| Position (HB) | -0.10 | -0.31 | 0.11 | .364 |
| Residence | 0.08 | -0.12 | 0.27 | .443 |

**Table S24.** Model predicting trust in the multiple-public goods game using the two-way

interaction of Public Good and Condition, including Ideology (centered),

Position, Gender, and Country of Residence as covariates. Marginal R^2^ is 0.10

and conditional R^2^ is 0.30. ‘PG’ represents the Public Good that participants

contributed to.

| Fixed Effect | β | Lower-95 | Higher-95 | *p*-value |
| --- | --- | --- | --- | --- |
| (Intercept) | 2.11 | 1.90 | 2.31 | <.001 |
| Ideology (centered) | -0.03 | -0.10 | 0.03 | .335 |
| PG (Unequal PG) | 1.68 | 1.66 | 1.70 | <.001 |
| Condition (A=B) | 1.00 | 0.97 | 1.02 | <.001 |
| Condition (A>B) | 1.75 | 1.73 | 1.77 | <.001 |
| Gender (male) | -0.05 | -0.23 | 0.13 | .583 |
| Gender (other) | 0.88 | 0.223 | 1.54 | .009 |
| Position (IB) | -0.03 | -0.24 | 0.19 | .820 |
| Position (HB) | -0.10 | -0.31 | 0.11 | .364 |
| Residence | 0.08 | -0.12 | 0.27 | .443 |
| Ideology (centered) * Condition (A=B) | -2.34 | -2.37 | -2.31 | <.001 |
| Ideology (centered) * Condition (A>B) | -3.97 | -4.00 | -3.94 | <.001 |

**Table S25.** Model predicting trust in the multiple-public goods game using the two-way

interaction of Ideology (centered) and Public Good, including Condition,

Position, Gender, and Country of Residence as covariates. Marginal R^2^ is 0.01

and conditional R^2^ is 0.21. ‘PG’ represents the Public Good that participants

contributed to.

| Fixed Effect | β | Lower-95 | Higher-95 | *p*-value |
| --- | --- | --- | --- | --- |
| (Intercept) | 3.16 | 2.95 | 3.37 | <.001 |
| Ideology (centered) | -0.05 | -0.11 | 0.02 | .196 |
| PG (unequal PG) | -0.42 | -0.44 | -0.41 | <.001 |
| Condition (equal PG = unequal PG) | -0.17 | -0.19 | -0.16 | <.001 |
| Condition (equal PG > unequal PG) | -0.24 | -0.25 | -0.22 | <.001 |
| Gender (male) | -0.05 | -0.23 | 0.13 | .583 |
| Gender (other) | 0.88 | 0.23 | 1.54 | .009 |
| Position (IB) | -0.03 | -0.24 | 0.19 | .820 |
| Position (HB) | -0.10 | -0.31 | 0.11 | .364 |
| Residence | 0.08 | -0.12 | 0.27 | .443 |
| Ideology (centered) * PG (unequal PG) | 0.02 | 0.01 | 0.03 | <.001 |

**Table S26.** Model predicting trust in the multiple-public goods game using the three-way

interaction of Ideology (centered), Public Good, and Condition, including

Position, Gender, and Country of Residence as covariates. Marginal R^2^ is 0.10

and conditional R^2^ is 0.31. ‘PG’ represents the Public Good that participants

contributed to.

| Fixed Effect | β | Lower-95 | Higher-95 | *p*-value |
| --- | --- | --- | --- | --- |
| (Intercept) | 2.11 | 1.90 | 2.31 | <.001 |
| Ideology (centered) | 0.01 | -0.06 | 0.08 | .804 |
| PG (Unequal PG) | 1.68 | 1.66 | 1.70 | <.001 |
| Condition (A=B) | 1.00 | 0.97 | 1.02 | <.001 |
| Condition (A>B) | 1.75 | 1.73 | 1.77 | <.001 |
| Gender (male) | -0.05 | -0.23 | 0.13 | .583 |
| Gender (other) | 0.88 | 0.23 | 1.54 | .009 |
| Position (IB) | -0.03 | -0.24 | 0.19 | .820 |
| Position (HB) | -0.10 | -0.31 | 0.11 | .364 |
| Residence | 0.08 | -0.12 | 0.27 | .443 |
| Ideology (centered)* PG (Unequal PG) | -0.12 | -0.14 | -0.11 | <.001 |
| Ideology (centered)*Condition (A=B) | -0.02 | -0.03 | 0.00 | .074 |
| Ideology (centered)*Condition (A>B) | -0.15 | -0.16 | -0.13 | <.001 |
| PG (Unequal PG)*Condition (A=B) | -2.34 | -2.37 | -2.31 | <.001 |
| PG (Unequal PG)*Condition (A>B) | -3.97 | -4.00 | -3.94 | <.001 |
| Ideology (centered)* PG (Unequal PG)* Condition (A=B) | 0.10 | 0.08 | 0.12 | <.001 |
| Ideology (centered)* PG (Unequal PG))* Condition (A>B) | 0.34 | 0.32 | 0.37 | <.001 |

## 3.3 Norms

**Table S27.** Model predicting normative expectations and descriptive expectations using

main effects, including the Position of the target, Gender, and Country of

Residence as covariates. Marginal R^2^ is 0.19 and 0.04 respectively, and

conditional R^2^ is 0.19 and 0.09, respectively. ‘PG’ represents the Public Good

that participants contributed to.

| Fixed Effect | β | Lower-95 | Higher-95 | *p*-value |
| --- | --- | --- | --- | --- |
| **Normative Expectations** | | | | |
| (Intercept) | 4.53 | 4.12 | 4.93 | <.001 |
| Ideology (centered) | -0.11 | -0.24 | 0.02 | .104 |
| PG (unequal PG) | -2.02 | -2.35 | -1.70 | <.001 |
| Target Position (IB) | 0.17 | -0.23 | 0.57 | .400 |
| Target Position (HB) | 0.58 | 0.18 | 0.98 | .005 |
| Gender (male) | -0.30 | -0.63 | 0.03 | .080 |
| Gender (other) | 0.98 | -0.85 | 2.81 | .300 |
| Residence | 0.19 | -0.14 | 0.52 | 0.262 |
| **Descriptive Expectations** | | | | |
| (Intercept) | 2.89 | 2.46 | 3.32 | <.001 |
| Ideology (centered) | 0.02 | -0.17 | 0.13 | .752 |
| PG (unequal PG) | -0.59 | -0.91 | -0.27 | <.001 |
| Target Position (IB) | 0.43 | 0.03 | 0.82 | .033 |
| Target Position (HB) | 0.63 | 0.24 | 1.03 | .002 |
| Gender (male) | 0.24 | -0.13 | 0.61 | .205 |
| Gender (other) | 2.00 | -0.05 | 4.05 | .057 |
| Residence | 0.42 | 0.05 | 0.79 | .028 |

**Table S28.** Model predicting normative expectations and descriptive expectations using the

interaction of Ideology (centered) and Public Good, including the Position of the

target, Gender, and Country of Residence as covariates. Marginal R^2^ is 0.19 and

0.05 respectively, and conditional R^2^ is 0.19 and 0.09, respectively. ‘PG’

represents the Public Good that participants contributed to.

| Fixed Effect | β | Lower-95 | Higher-95 | *p*-value |
| --- | --- | --- | --- | --- |
| **Normative Expectations** | | | | |
| (Intercept) | 4.53 | 4.12 | 4.93 | <.001 |
| Ideology (centered) | -0.20 | -0.39 | -0.02 | .031 |
| PG (unequal PG) | -2.03 | -2.35 | -1.70 | <.001 |
| Target Position (IB) | 0.17 | -0.23 | 0.57 | .400 |
| Target Position (HB) | 0.58 | 0.18 | 0.98 | .004 |
| Gender (male) | -0.30 | -0.63 | 0.03 | .079 |
| Gender (other) | 0.98 | -0.85 | 2.81 | .294 |
| Residence | 0.19 | -0.14 | 0.52 | .262 |
| Ideology (centered)* PG (unequal PG) | 0.19 | -0.07 | 0.44 | .155 |
| **Descriptive Expectations** | | | | |
| (Intercept) | 2.89 | 2.46 | 3.31 | <.001 |
| Ideology (centered) | -0.19 | -0.38 | 0.01 | .060 |
| PG (unequal PG) | -0.59 | -0.91 | -0.27 | <.001 |
| Target Position (IB) | 0.43 | 0.04 | 0.82 | .032 |
| Target Position (HB) | 0.63 | 0.24 | 1.03 | .002 |
| Gender (male) | 0.24 | -0.13 | 0.61 | .205 |
| Gender (other) | 2.00 | -0.05 | 4.05 | .057 |
| Residence | 0.42 | 0.05 | 0.79 | .028 |
| Ideology (centered)* PG (unequal PG) | 0.33 | 0.08 | 0.58 | .011 |

# 4 Supplement References

1. Gill, D. & Prowse, V. A structural analysis of disappointment aversion in a real effort competition. *Am. Econ. Rev.* **102**, 469–503 (2012).

2. Hoenig, L. C., Pliskin, R. & De Dreu, C. K. W. Equality and efficiency shape cooperation in multiple public good provision problems. *PsyArXiv* (2023).

3. Murphy, R. O., Ackermann, K. A. & Handgraaf, M. J. J. *Measuring Social Value Orientation*. *Judgment and Decision Making* vol. 6 (2011).
